# Exercise mimetics: a novel strategy to combat neuroinflammation and Alzheimer’s disease

**DOI:** 10.1186/s12974-024-03031-9

**Published:** 2024-02-02

**Authors:** Renqing Zhao

**Affiliations:** College of Physical Education, Yangzhou University, Yangzhou, China

## Abstract

Neuroinflammation is a pathological hallmark of Alzheimer’s disease (AD), characterized by the stimulation of resident immune cells of the brain and the penetration of peripheral immune cells. These inflammatory processes facilitate the deposition of amyloid-beta (Aβ) plaques and the abnormal hyperphosphorylation of tau protein. Managing neuroinflammation to restore immune homeostasis and decrease neuronal damage is a therapeutic approach for AD. One way to achieve this is through exercise, which can improve brain function and protect against neuroinflammation, oxidative stress, and synaptic dysfunction in AD models. The neuroprotective impact of exercise is regulated by various molecular factors that can be activated in the same way as exercise by the administration of their mimetics. Recent evidence has proven some exercise mimetics effective in alleviating neuroinflammation and AD, and, additionally, they are a helpful alternative option for patients who are unable to perform regular physical exercise to manage neurodegenerative disorders. This review focuses on the current state of knowledge on exercise mimetics, including their efficacy, regulatory mechanisms, progress, challenges, limitations, and future guidance for their application in AD therapy.

## Introduction

As our society continues to age, we face more challenges from diseases and healthcare costs, especially neurodegenerative disease [1, 2]; for example, Alzheimer’s disease (AD) has affected millions of people worldwide and caused a tremendous burden to patients and society [3, 4]. AD seriously impacts brain health and quality of life [3, 5], but effective treatment for the disease is currently lacking. Fortunately, growing evidence has revealed that a physically active lifestyle confers exercise partners with multi-organ benefits [6, 7], particularly improving brain plasticity and function [8, 9]. For example, several studies have shown that exercise can enhance various aspects of brain function such as cognitive function, memory, and learning [10–12], providing therapeutic protocols for neurodegeneration and cognitive impairment in AD. Exercise can bring many benefits for AD pathology including decreasing amyloid-beta (Aβ) formation, aggregation, and clearance and inhibiting tau hyperphosphorylation [13–15]. The amelioration of those pathological profiles is associated with several modifications in the brain, such as neuroinflammation, oxidative stress, and synaptic dysfunction [15–17]. By improving pathological conditions, exercise restores impaired hippocampal neurogenesis, synaptic degradation, and neurotransmission and enhances cognitive function and mood [11, 16, 18].

The beneficial effects of exercise on AD pathology are believed in part through its capacity to generate a range of molecules such as brain-derived neurotrophic factor (BDNF), clusterin (CLU), and irisin that can modify microglial activation, suppress oxidative stress, and reduce neuroinflammation [17, 19–21]. Recent studies have revealed that exercise stimulates peripheral organs generating various factors that can enter the bloodstream and pass through the blood–brain barrier (BBB) where they affect neurogenesis, synaptic plasticity, neuronal networks, and various neurogenic factors [14, 21–24]. Accordingly, several lines of evidence have suggested that muscle is not only an exercising organ, but an endocrine tissue producing numerous cytokines that target remote organ function [25–27]. For example, exercise promotes muscle to secret fibronectin type III domain-containing protein 5 (FNDC5)/irisin, a peroxisome proliferator-activated receptor gamma coactivator 1-alpha (PGC-1α) dependent myokine, which promotes synaptic plasticity, reduces oxidative stress and ameliorates neuroinflammation in AD rodent models [16, 19, 28], proposing a connection between muscle and brain. Additionally, the liver generates a large number of factors such as glycosylphosphatidylinositol (GPI)-specific phospholipase D1 (Gpld1) and S-adenosylmethionine (SAM) that are important for metabolism and neuroinflammation and can cross BBB affecting brain function in transgenic murine AD models, revealing a liver–brain axis [22, 29–31]. Those findings suggest that there exists a close communication between exercise–peripheral tissue–brain and highlight the regulatory role of exercise molecules in mediating the function of the central nervous system (CNS). Those molecules not only regulate exercise effects, but also affect various aspects of brain function. Therefore, they have the potential to provide novel approaches for brain disorders like AD. Recently, several studies have explored the potential of the administration of those exercise factors to mimic the exercise effects on brain function. One study transferred the “runner plasma” derived from exercising rodents to sedentary controls finding that it brought many benefits for AD brains including neurogenesis, synaptic plasticity, and memory [22]. Subsequent analysis confirmed Gpld1 accounting for this effect [22]. Similarly, another study revealed that 28 days of voluntary running significantly enhanced clusterin levels, and “runner plasma” containing high levels of clusterin showed significant effects on brain function in AD rodent models [21]. Currently, a lot of exercise molecules have been tested in different animal models and they transfer, to different extent, the exercise effects seen in exercise interventions [32–34]. It is suggested that exercise mimetics could be a promising approach for brain health in various brain disorders. In addition, despite the obvious benefits induced by exercise, participation in physical exercise is extremely low, either due to the difficulty in performing regular exercise or lack the interest. Therefore, pharmacological intervention, such as exercise mimetics, could be a potential alternative strategy. Exercise mimetics are pharmacologic compounds that play a key role in exercise-induced beneficial effects. Although a range of molecules has been tested and proven effective in improving brain health, there are still a large number of questions required to be addressed, for instance, the underlying mechanism, optimal dose, treatment duration, and side effects. Moreover, there are also some compounds that did not show favorable effects as expected, for instance, the use of some anti-inflammation agents to treat AD [35]. Therefore, this review focuses on the current state of knowledge regarding the challenges and opportunities of exercise mimetics, including their efficacy, regulatory mechanisms, progress, challenges, limitations, and future guidance for their application in AD therapy.

## Neuroinflammation is a new hallmark of AD pathology

Aβ and tau are two critical proteins that aggregate abnormally in the brain of AD patients or individuals with mild cognitive impairment (MCI), leading to neurodegeneration and cognitive impairment. Under the pathological conditions of AD, the amyloid precursor protein (APP) is first cleaved by the beta site APP cleaving enzyme 1 (BACE1) and subsequently primed by γ-secretase, releasing Aβ and stimulating the amyloidogenic pathway [36]. When the production of Aβ exceeds the capacity of clearance (such as transportation into the blood vessels and local degradation by microglia), Aβ monomers begin to aggregate by combining with each other to form different complexes, such as oligomers, polymers, and insoluble fibrils, which are regarded as danger-associated molecular patterns (DAMPs) and is able to cause neuroinflammation, synaptic dysfunction and neuronal degradation in AD [37]. However, the role of Aβ in the pathogenesis of AD and cognition impairment has been challenged by the findings that clinical treatment with Aβ antibodies has been shown to successfully reduce Aβ deposition in the brain, but not to improve the cognitive function of patients [38]. In consistency with these findings, only about a third of people with Aβ accumulation eventually develop AD after 70 years of age, while over half of the population remains cognitively intact for their entire life [39, 40], indicating Aβ in the CNS may not always be neurotoxic [41, 42]. Recent evidence has suggested that cognitive impairment may be more related to neuronal loss and dysfunction induced by tau hyperphosphorylation instead of Aβ [43]. Tau protein normally helps to stabilize microtubules in neurons, but in AD it becomes abnormally phosphorylated and forms neurofibrillary tangles (NFTs) causing tau aggregation and neurotoxic [44]. Emerging evidence suggests that the neurotoxic effects of tau in AD may be linked to its interaction with neuroinflammation [45]. Neuroinflammation has been identified as a key driver of AD and other neurodegenerative diseases in tau pathology [46–48]. Recently, a study has revealed that dense-core plaques are formed after reactive microglia engulf amorphous Aβ plaques to reduce inflammation [49]. Moreover, long-term exposure to various stimuli, including DAMPs and pathogen-associated molecular patterns (PAMPs) which are signals of internal or external brain injury, can trigger neuroinflammation by activating immune reactions and causing inflammatory cascades that are closely associated with neurodegeneration in AD [50]. Together, current evidence indicates that neuroinflammation is a hallmark of AD pathology.

## Neuroinflammation activation

Neuroinflammation is the term describing the inflammatory reaction in CNS when exposed to various harmful stimuli, such as infection, toxins, misfold protein, and ischemia [47]. This process involves the reactive immune cells in the brain, such as microglia and astrocytes, which release various inflammatory molecules, including interleukin (IL)-1β, IL-6, and tumor necrosis factor-alpha (TNF-α), and recruit other immune cells. Additionally, reactive oxygen species (ROS) and chemokines such as C–C motif chemokine ligand 1 (CCL1) and C–X–C motif chemokine ligand 1 (CXCL1) are also produced by immune cells in the brain [51]. Microglia are the key innate immune cells in the brain that are engaged in neuroinflammation [51, 52]. In the context of inflammation, certain inflammatory cytokines such as TNFα, IL-6, and IL-1β can activate the transport systems of BBB and make it more permeable, allowing other cells like capillary endothelial cells and blood cells to infiltrate the BBB and contribute to neuroinflammation and neurodegeneration [53–55]. Additionally, microglia can interact with astrocytes to regulate neuroinflammation [56]. Astrocytes play a key role in maintaining brain health by controlling blood flow, preserving BBB, and supporting synapses and neurotransmitters [57]. Astrocytes can induce neuroinflammation which is associated with the degenerative progression of tauopathy [58]. When microglia are reactive, they release inflammatory molecules such as IL-1β, TNF-α, and complement, which can cause astrocytes to adopt a pro-inflammatory state [59]. On the other hand, in conditions such as AD, astrocytes can influence microglial activation by releasing IL-3, which promotes the clearance of phosphorylated tau and NFTs by microglia [60]. Neuroinflammation can have both positive and negative effects on the CNS, depending on the type, duration, and intensity of the inflammatory response [47, 61]. As an adaptive mechanism, neuroinflammation aids in pathogen elimination, tissue repair, and clearance of cellular debris, while also modulating synaptic plasticity and neuronal activity [62, 63]. For example, some pro-inflammatory cytokines, such as IL-1β and TNF-α, can enhance synaptic plasticity and memory function under physiological conditions [64]. However, prolonged neuroinflammation can be harmful, leading to synaptic loss, neuron death, and neurogenic inhibition [65, 66]. For example, under pathological conditions, IL-1β contributes to increased ROS production, damaging cellular components and causing neuronal death, while IL-6 induces the expression of pro-apoptotic genes like Bax and caspase-3, triggering programmed cell death in neurons [64]. Generally, neuroinflammation in neurodegenerative diseases, such as AD, often develops a prolonged process that seems not to be resolved by itself and is regarded as a critical contributor to neurodegeneration [67].

**Fig.1:**
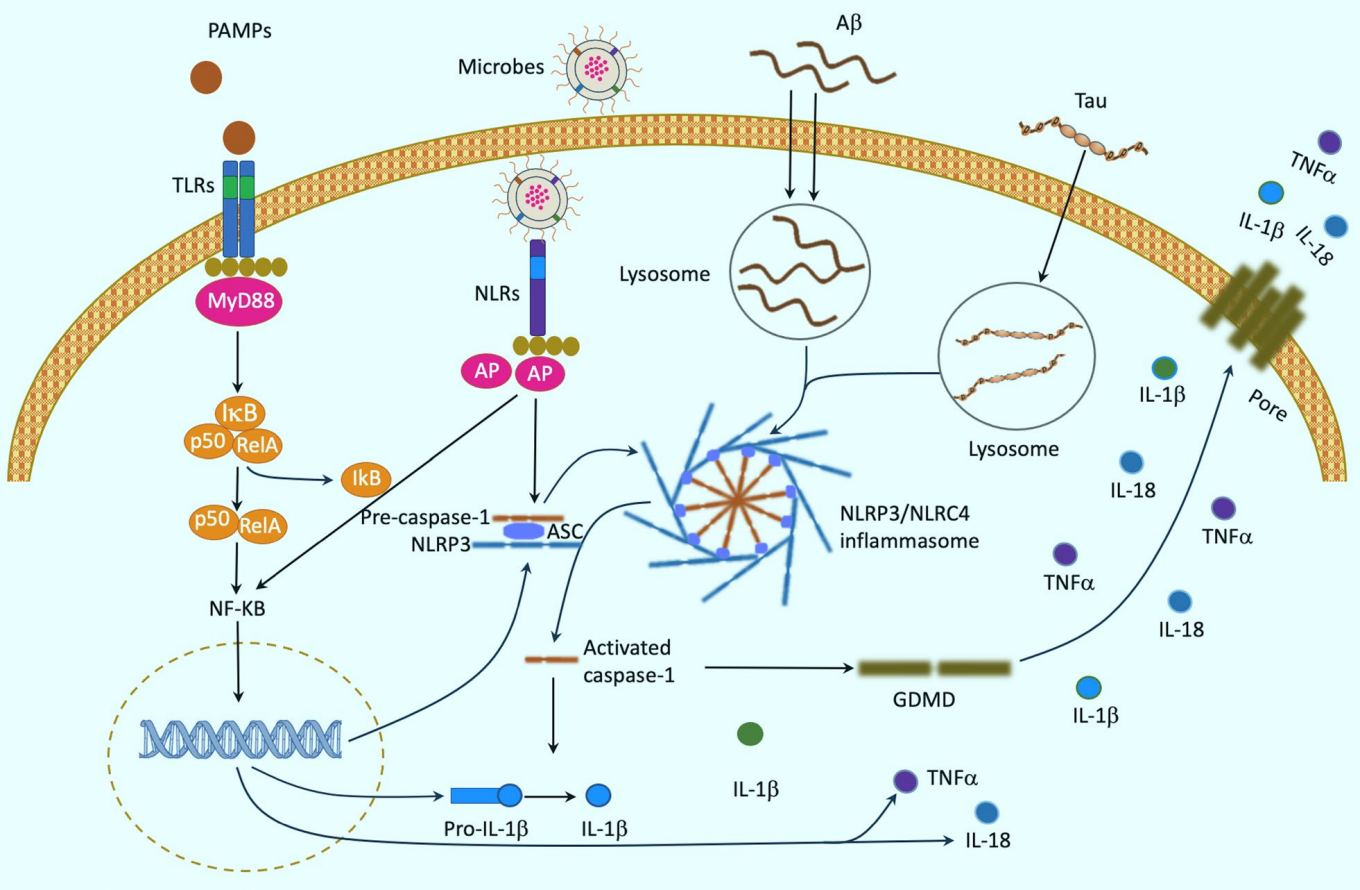
Neuroinflammation activation. TLRs are a family of membrane-bound receptors that recognize and bind to PAMPs. Upon binding to PAMPs, TLRs recruit adaptor molecules such as MyD88, that form a molecular complex called Myddosome. The complex activates a subsequent kinase cascade, which leads to the release of NF-κB from the cytoplasm and translocation to the nucleus, where it regulates the expression of genes involved in inflammation, such as IL-1β and TNF-α. NLRs are a group of cytosolic receptors and sense DAMPs and PAMPs that enter the cell. When they detect pathogens, NLRs undergo oligomerization and recruit ASC to assemble the NLRP3 or NLRC4 inflammasome. The filamentous ASC then attracts pro-caspase 1, which becomes activated and cleaves pro-IL-1β into mature cytokines. Additionally, in the context of AD, Amyloid aggregation and Tau hyperphosphorylation can facilitate the formation of NLRP3 inflammasome leading to microglial activation. The interaction between Aβ and microglia activation is involved in diverse molecular signaling such as the TREM2–TYROBP axis. AP, adaptor proteins; DAMPs, danger-associated molecular patterns; PAMPs, Pathogen-associated molecular patterns; TLRs, Toll-like receptors; TNF-α, Tumor necrosis factor-alpha; ASC, Apoptosis-associated speck-like protein containing a CARD; NLRP3, NOD-like receptor family pyrin domain containing 3; NLRPC4, NOD-like receptor family CARD domain-containing protein 4; AD, Alzheimer's disease; Aβ, amyloid beta; TREM2, triggering receptor expressed on myeloid cells 2; TYROBP, tyrosine kinase binding protein; GDMD: gasdermin D

The activation of innate immune response occurs through the recognition and combination of ligands with pattern recognition receptors (PRRs), such as Toll-like receptors (TLRs) and nucleotide oligomerization domain (NOD)-like receptors (NLRs)[46] (Fig. 1). Immune signaling receptors can facilitate important functions like phagocytosis and reactive oxygen production, which ultimately lead to the elimination of pathogens. TLRs are a class of membrane-bound receptors that recognize PAMPs. Upon binding to PAMPs, TLRs recruit adaptor molecules such as MyD88 that form a molecular complex called Myddosome. The complex activates a subsequent kinase cascade, which leads to the release of nuclear factor kappa B (NF-κB) from the cytoplasm and translocation to the nucleus, where it regulates the expression of genes involved in inflammation, such as IL-1β and IL-18 [68]. Additionally, NLRs are a group of cytosolic receptors that mediate post-transcriptional events upon sensing a specific trigger [69–71] (Fig. 1). Upon activation, NLRs can initiate divergent signaling pathways, depending on the specific N-terminal domain and downstream effectors recruited [69]. Some NLRs like NOD1 and NOD2 recognize peptidoglycan fragments from bacteria, which can trigger the NF-κB and mitogen-activated protein kinase (MAPK) pathways, leading to the production of pro-inflammatory cytokines and chemokines [72]. Other NLRs, such as the NOD-like receptor family pyrin domain containing 3 (NLRP3) and NLR family CARD domain-containing protein 4 (NLRC4), can detect various PAMPs and DAMPs, including crystals, cell debris, toxins, oxidative stress, and bacterial flagellin [69]. Upon activation, they form a complex with apoptosis-associated speck-like protein containing a CARD (ASC) and pro-caspase-1, which triggers the activation of caspase-1 and the conversion of pro-IL-1β and pro-IL-18 into mature cytokines [69, 71, 73]. Additionally, in the context of AD, amyloid aggregation and Tau hyperphosphorylation can facilitate the formation of NLRP3 inflammasome, which increases the activation of microglia, in part, by mediating some molecular signaling like the triggering receptor expressed on myeloid cells 2 (TREM2) pathway [74].

## Microglia and AD pathology

### Microglia: morphology, function, and role in neurodegeneration

**Fig. 2:**
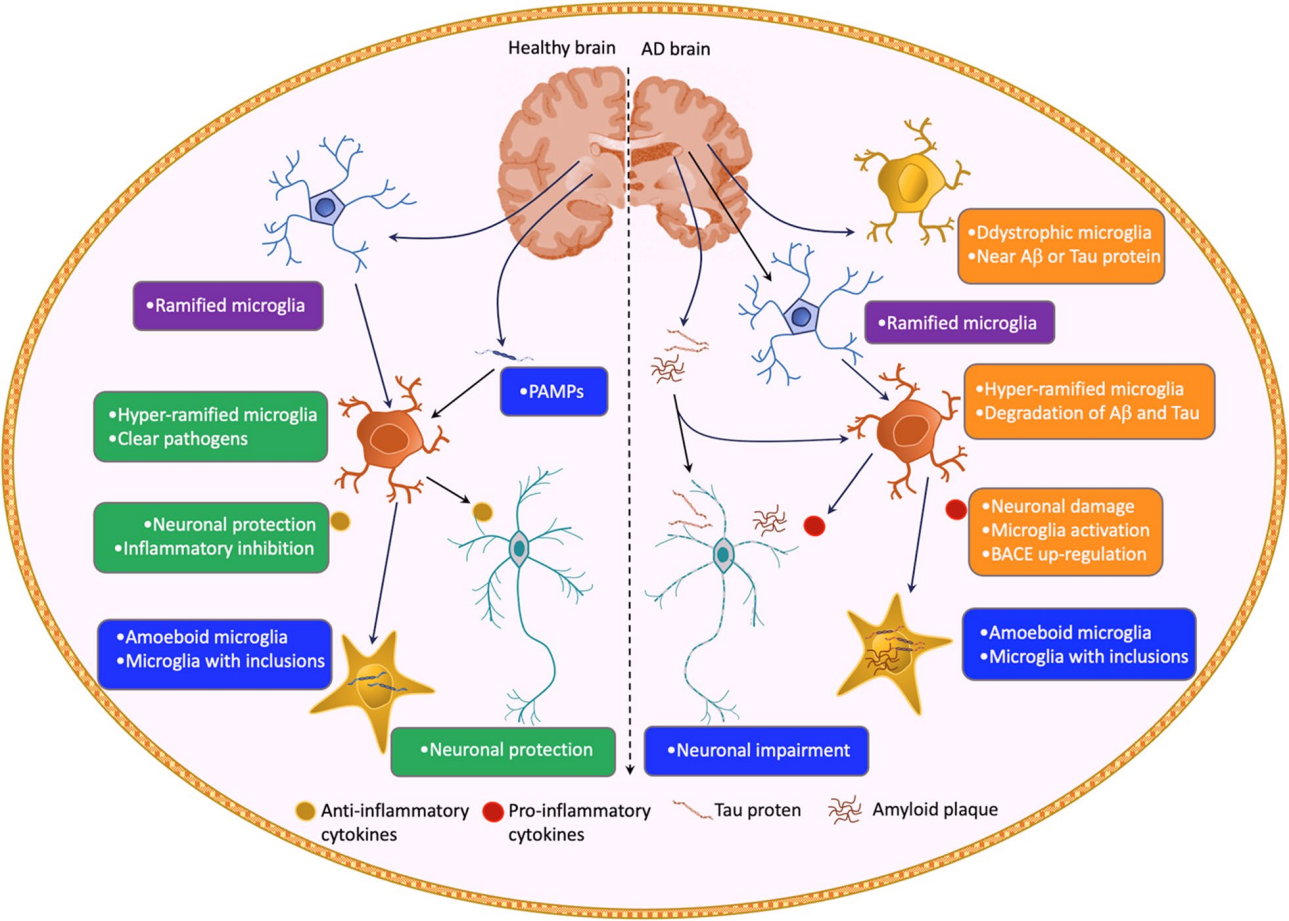
Microglial function and AD pathology. Microglia are present throughout the brain and their distribution varies by region. They have different shapes that reflect their essential functions of maintaining cerebral homeostasis and defense. Ramified microglia are the most common type in the healthy brain. They use branches to monitor the cerebral environment and detect injury signals. They then move their branches toward the damaged site and trigger a microglial response that involves reshaping synapses and keeping myelin stable. Microglia become highly ramified and have a robust power to clear pathogens. The highly branched form of microglia can also transform into a less branched shape as a result of engulfing pathological items, such as Aβ and Tau proteins. Microglia become dystrophic and hyperinflammatory with aging and neurodegenerative disorders such as AD. Moreover, the morphology of microglia varies in different regions and stages of the diseased brain. The temporal changes of the microglia aspect could depend on the intensity and duration of exposure to the harmful environment but could also be attributed to the divergent reaction of microglia to differing substances like Aβ or tau. AD, Alzheimer’s disease; Aβ, amyloid beta; PAMPs, Pathogen-associated molecular patterns

Microglia, the resident innate immune cells in the CNS, have long been implicated in the early response to pathological stimuli [67, 75]. They are distributed throughout the brain and account for 5–12% of all brain cells in mice, varying by region [76]. They have different shapes which reflect their key functions of maintaining cerebral homeostasis and defense (Fig. 2). Ramified microglia are the most common type in the healthy brain that utilize their branches to constantly monitor the cerebral environment and detect injury signals. They then move their branches toward the damaged site and trigger a microglial response that involves reshaping synapses and keeping myelin stable [77, 78]. Subsequently, microglia become highly ramified and have a robust power to clear pathogens. However, the highly branched form of microglia can also transform into an amoeboid shape when they encounter and engulf pathological stimuli [77, 79]. Microglia in older brains have fewer branches and cover less area, which may affect their ability to perform their immune functions [80–83]. Moreover, microglia become dystrophic and hyperinflammatory with neurodegenerative disorders [84]. Intriguingly, microglia aspects also vary in different regions and stages of the diseased brain. For example, microglia near amyloid plaques change a lot in their shape and function, while microglia far from plaques alter little over time [85]. Furthermore, microglia in the later stages of the disease often experience dramatic morphological changes compared to those in earlier stages [86]. The temporal changes of the microglia aspect could depend on the intensity and duration of exposure to the harmful environment [87] but could also be attributed to the divergent reaction of microglia to differing substances like Aβ or tau [88]. The phenotypic changes of microglia are believed to be caused in part by hyperphosphorylated tau leading to inhibition of immunosurveillance function and driving the disease development via forming more tangles in the brain [86]. To facilitate the identification of microglial function, researchers have formulated a simplified model of microglia activation, with M1 referring to the type of pro-inflammation and M2 referring to the type of anti-inflammation [89]. Growing evidence has challenged this simplified paradigm and revealed that microglia do not always fit into the M1–M2 categories [67, 90]. However, the M1–M2 dichotomy remains commonly applied in describing the notion that microglia can be helpful or harmful when they react to pathogens.

### Transcriptomic profiles of microglia revealed by scRNA-seq and snRNA-seq in neuroinflammation

The investigation of neuroimmune communication in the brain has been dramatically advanced by the application of single-cell RNA sequencing (scRNA-seq) and single-nucleus RNA sequencing (snRNA-seq) techniques. Mapping single-cell or single-nucleus transcriptomes has provided novel insights into the cellular and molecular characteristics of macroglia–neuroinflammation interactions. This approach has revealed unexpected details such as the identification of new cell populations, subpopulations, and states. It has also uncovered the spatial and temporal patterns of gene expression and has shed light on the molecular pathways and underlying mechanisms of neuroinflammation and neurodegeneration [91]. Using transcriptional single-cell profiling, Keren-Shaul et al. [92] identified a novel subcluster of disease-associated microglia and revealed their trajectory across AD progression. The transition from a homeostatic stage to a disease-related phase was hallmarked by expressing a unique set of genes, which enhanced their capacity for metabolizing lipids and engulfing plaques in transgenic AD mice [92]. This study confirmed that the Tyrobp and TREM2 formed a signaling complex, linked to Aβ removal, that could increase the phagocytic activity of microglia and contribute to the inhibition of inflammatory reaction via suppressing microglia-regulated cytokine release [93]. Additionally, snRNA-seq profiling of postmortem older human brains revealed aging-associated phenotypes of microglia, highlighting the absence of a set of genes involved in actin assemblies, such as Talin-1, Profilin-1 and vasodilator-stimulated phosphoprotein (VASP), indicating a decreased capacity of cellular motility and migration toward the damaged region [94]. Furthermore, genes related to axonal guidance, cell adhesion, and the sensome, such as IL6R, TLR10, intercellular adhesion molecule (ICAM)-3, semaphorin (SEMA)3C and SEMA7A also decreased, indicating the impairment of microglial sensing with aging [94]. Using snRNA-seq protocol, Mathys et al. [95] tracked microglial activation along with the disease process in the CK-p25 mouse model with severe neurodegeneration. They discovered distinct microglia cell states during the progression of neurodegeneration. An early-reaction state was found to express cell-cycle genes and genes related to DNA replication and repair, while a late-reaction state expressed interferon response and anti-virus genes [95]. However, the early and late microglia states are related, as some transcripts that increase in late microglia are already upregulated in early microglia. This suggests that early microglia are a transient intermediate phase that reflects the cellular reprogramming of homeostatic microglia in reaction to the neurodegeneration [95]. Together, transcriptomic profiling datasets have enabled the identification of new subclusters of reactive microglia that represent distinct activation states previously unreported [96]. These transcriptomic studies have helped establish a molecular atlas of microglial-mediated neuroinflammation and its relation to neurodegeneration [97].

### The interaction between microglial activation and AD pathology

Reactive microglia show a close spatial relationship with amyloid plaques in the brains of AD patients [98, 99]. Moreover, both postmortem and in vivo neuroimaging studies have revealed a correlation between the amount of pathological tau and the number of microglial cells in AD brains, suggesting that microglia interact with the key pathological features of AD [100, 101]. The interplay among amyloid plaques, microglia activation, and NFTs emerges as a key area of research in AD pathology. Early studies have revealed that distinct Aβ species, such as small and large Aβ oligomers, could cause different patterns of microglial activation and the production of pro-inflammatory (such as IL-1β, IL-6, and TNF-α) or anti-inflammatory (such as TGF-β) cytokines, as well as chemokines, diverse cell adhesion molecules, and ROS, all of which could modify neuronal morphology and function [102–105]. Microglia can recognize different Aβ species through various PRRs [106, 107], such as TLRs, NLRs, TREM2, CD14, CD33, and scavenger receptors (SR). These receptors can activate different molecular pathways that induce pro-inflammatory, anti-inflammatory, or phagocytic changes in the microglia [106–112].

Among multiple pathways regulating the interaction between microglial activation and AD pathology, one signaling is involved in the activation of NLRP3 inflammasome, a protein complex that triggers inflammatory reactions in microglia. The activation of this complex is stimulated by NF-κB [113], which leads to a conformational change and subsequent inflammasome assembly leading to the release of caspase 1 and IL-1β [114, 115]. Additionally, TREM2, known as a genetic risk factor for AD, is another key molecule that mediates Aβ-induced oxidative stress and inflammatory response in microglia [116, 117]. The upregulated TREM2 was observed on Aβ-triggered microglia in AD patients and transgenic AD murine models [118]. The TREM2 cascades are involved in promoting microglial proliferation and phagocytosis, as well as the release of pro-inflammatory factors, microglial metabolism, and survival [119, 120]. Reactive microglia have the ability to phagocytose Aβ plaques, but their degradation capacity is limited due to the slow rate of autophagy, raising concerns about the effectiveness of microglia in the Aβ clearance [121]. The efficacy of microglia in clearing Aβ aggregations remains unclear because microglia in later-stage AD brains have shortened branches, shrunken coverage areas, and fragmented processes, indicating surveillant dysfunction [88, 122] (Fig. 2). Additionally, the expression of TREM2 and CD68 on plaque-related microglia is downregulated in AD patients, indicating an impaired phagocytic function of microglia [123]. Interestingly, a similar microglial change was also seen in aging individuals, hinting at the contribution of dysfunctional microglia in neurodegeneration [124, 125]. Moreover, microglia can release cytokines like IL-1β, IL-6, TNF and IFNγ that boost β-secretase production, an enzyme that increases harmful Aβ generation through the NF-κB pathway [126, 127]. Generally, microglia have a complex role in AD that varies depending on the disease stage and pathology context. They can prevent the spread and seeding of amyloid plaques, but this may also increase the neurotoxicity of the plaques [128]. On the other hand, they can also accelerate the formation of amyloid plaques through ASC particles [129]. Given the toxic nature of soluble amyloid species, this process may be helpful by depositing amyloid [129].

Exposure to tau oligomers and fibrils in vitro is sufficient to change microglial morphology and cytokine production [130]. Subsequent experiments applying rodent tauopathic models verified that microglia stimulated by tau in vivo show increased expression of genes related to disease-associated microglia (DAM), such as phagocytic and inflammatory genes [131]. Microglia can internalize tau fibrils in cell cultures as well as healthy rodent brains [132]. The process involves the activation of the complement system through both classical and alternative pathways [131]. However, the inspection of pathological alterations in human brains indicated the dysfunction of microglia, demonstrating tau pathology-induced morphological degradation [86]. The deleterious alterations are assumed to stem from the senescence of microglia and amyloid toxicity and the promotion of the diffusion of the tau pathology [133]. Aβ can cooperate with microglia to facilitate tau pathology [134]. Upregulation of NLRP3 in microglia facilitates the harmful association between Aβ and tau pathology, and the enhanced tau pathology is likely to help clear the Aβ [135]. The divergent microglial response to different stimuli may account for the complicated interactions. A pivotal microglial signaling pathway, the TREM2–TYROBP axis, could intercommunicate with both Aβ_42_ and tau protein signaling to regulate the genic expression [74]. Almost half of TREM2–TYROBP mediated pathways activated by Aβ overlap with the pathways triggered by tau, hinting that microglia acts as a co-regulator for the Aβ-tau interaction [74]. Together, those observations might propose that there exists a complex communication between microglia, amyloid plaque, and Tau protein in AD.

## Astrocytes and AD

Reactive gliosis is a major component of neuroinflammation that occurs when astrocytes are exposed to various pathological stimuli, such as mechanical damage, ischemia, or abnormal protein aggregation [136]. This process transforms astrocytes into “reactive astrocytes” with hypertrophic processes and increased glial fibrillary acidic protein (GFAP) expression. Reactive astrocytes are heterogeneous and have different phenotypes and functions depending on the type and severity of the inflammatory stimulus. A1 and A2 astrocytes are two contrasting phenotypes of reactive astrocytes. The NF-κB pathway induces A1 astrocytes, which are “harmful” because they secrete inflammatory factors that disrupt homeostasis and cause neuronal and oligodendrocyte death [137, 138]. Conversely, the signal transducers and activators of transcription 3 (STAT3) pathway induces A2 astrocytes, which are “protective” because they secrete neurotrophic factors that help restore homeostasis [137]. However, recent studies challenge the binary model of A1 and A2 astrocytes and suggest that astrocytes may have multiple activation states that vary according to the neural circuits and brain regions they belong to [139]. Astrocytes are involved in the pathological processes of AD [140]. Astrocytes near amyloid plaques can engulf amyloid granules [141, 142] and degrade Aβ aggregates [142]. However, A1 astrocytes, which are harmful, are prevalent in the brains of individuals with AD, indicating their negative role in the disease [138]. Moreover, reactive astrocytes have been observed to produce abnormal amounts of GABA and glutamate in rodent models of AD, resulting in memory deficits and synaptic degradation [143, 144]. Additionally, reactive astrocytes can compromise the integrity of the BBB, allowing blood-derived DAMPs or PAMPs to enter the damaged BBB, leading to immune responses and promoting the accumulation of Aβ and disease progression [145, 146]. Importantly, reactive astrocytes may facilitate the formation of the earliest amyloid plaques [147], and interact with microglia to mediate the detrimental effects of microglia in the AD progression [138].

## Exercise and AD

### Therapeutic implications of exercise for AD

**Fig. 3:**
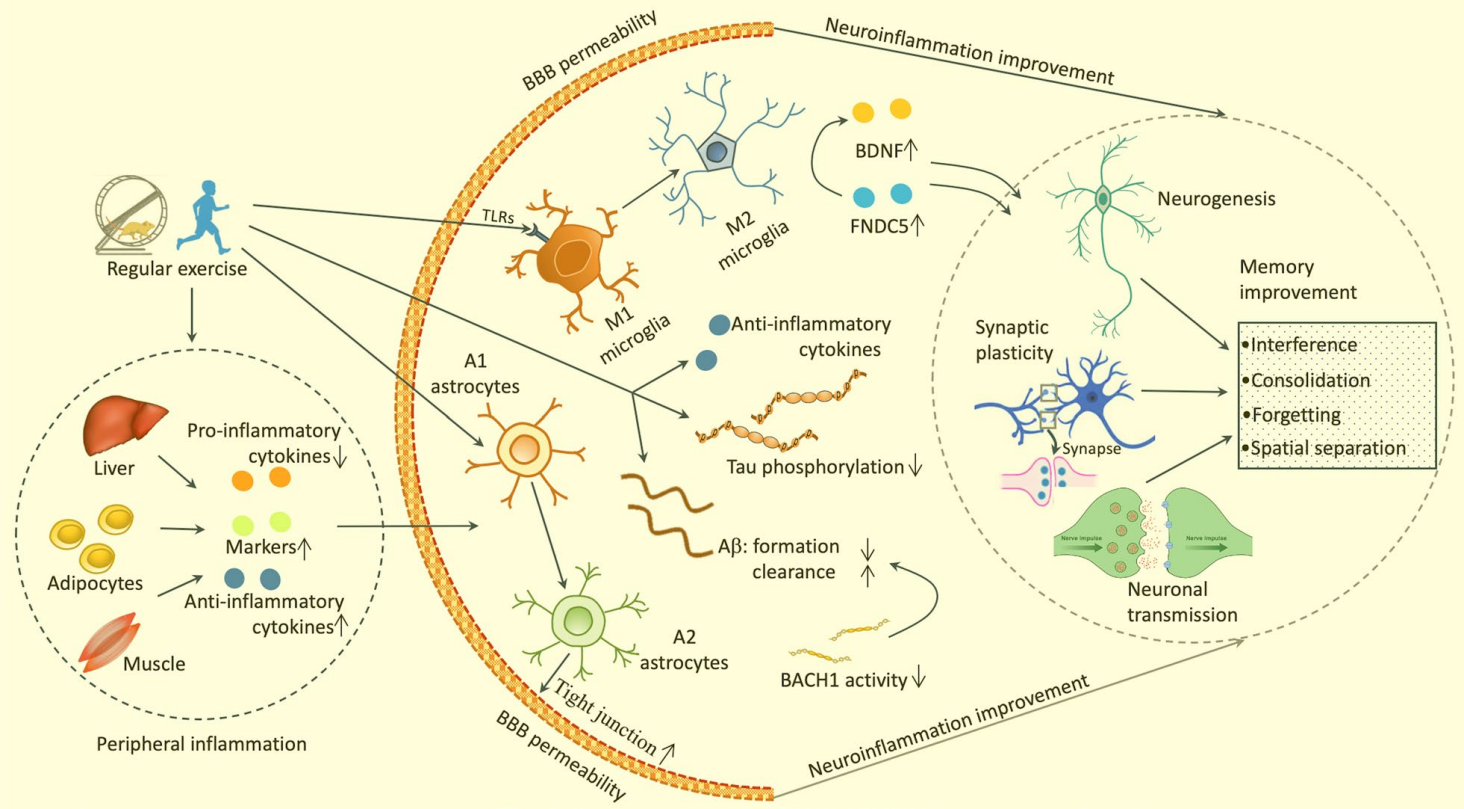
Exercise for neuroinflammation and AD. Physical exercise can exert multiple positive effects on the brain of AD, such as enhancing cerebral blood flow, neurogenesis, synaptic plasticity, neurotrophic factors, antioxidant defense, and cognitive function. Exercise can inhibit the formation and deposition of Aβ and abnormal phosphorylation of Tau, partly by affecting α- and γ-secretase activity, BDNF production, and BACE1 function. More importantly, physical exercise can modulate neuroinflammation by directly and indirectly mediating the immune response of the CNS. Physical exercise can impact the activation state and phenotype of microglia and astrocytes in AD, resulting in the shift of the polarization of microglia and astrocytes from a pro-inflammatory (M1 or A1) to an anti-inflammatory (M2 or A2) pattern. This immune action results in reduced production of pro-inflammatory cytokines and enhanced production of anti-inflammatory molecules. Furthermore, physical exercise can suppress the activation of inflammasomes, such as NLRP3, which in turn decreases the production of IL-1β and caspase-1. Additionally, physical exercise can strengthen the thigh connection of the BBB, which can prevent the infiltration of peripheral immune cells and inflammatory molecules into the brain. AD, Alzheimer’s disease; Aβ, amyloid beta; BACE1, beta site APP cleaving enzyme 1

As current pharmacological treatments for AD have limited clinical efficacy, researchers are prompted to explore alternative strategies to prevent or treat this disease (Fig. 3). The latest report on AD in 2022 has proposed a range of risk factors for this disease, particularly the lack of exercise [148]. Recent evidence has suggested that sedentary behavior is associated with reduced cognitive function in individuals with AD, which underscores the importance of physical exercise for preventing the disease [149]. A population-based study encompassing 160,00 subjects showed that individuals who exercised regularly had a 45% lower risk of developing AD [150]. A comparable result (with a 53% reduction in AD risk for active people) was observed in a longitudinal study of 716 older people on the relationship between physical activity and dementia risk [151]. These findings have provided robust evidence to support the preventive role of physical exercise in AD. Regular exercise can reduce risk factors of AD, such as obesity, hyperlipidemia, hypertension, and type 2 diabetes mellitus (T2DM) [152]. Furthermore, exercise also shows various favorable effects for alleviating AD components in observational human and experimental animal studies [153]. Although there are some inconsistencies in the findings [154, 155], aerobic exercise has been found to enhance executive functions, memory, and cognitive performance in people with mild MCI [156–158]. Exercise can also enhance learning and memory function by enhancing adult neurogenesis in the hippocampus of rats with brain injury [159], indicating the potential of exercise in maintaining cerebral plasticity and function. Similarly, voluntary wheel exercise for 5 months was found to significantly reduce the levels of Aβ40 and Aβ42 in the transgenic murine brain [13], and a significant reduction in amyloid deposition and tau phosphorylation was observed in APP/PS1 transgenic mice following a 5-month treadmill running [160]. Exercise-related beneficial effects on AD pathology are associated with its capacity to affect Aβ deposition by regulation of α- and γ-secretase activity [161]. Additionally, 1 month of voluntary wheel exercise has been found to significantly increase BDNF levels in the rodent hippocampus by stimulating lactate secretion in skeletal muscle, which, in turn, enhances neuronal function and memory capacity [162]. BDNF has also been shown to reduce the activity of BACE1, a key enzyme responsible for cleaving APP into Aβ peptides, which may help inhibit the development of AD [163]. Interestingly, recent evidence has shown that exercise can affect immune function in AD murine models [164], indicating that anti-inflammation may play a role in the exercise-induced benefits of AD. Since AD is recognized as a neuroinflammatory disease, exercise may impact the pathology of AD by mediating the inflammatory status.

### Exercise alleviates inflammation

Exercise is a vital component of human health and well-being, especially generating multiple benefits for the immune system, such as enhancing cell survival, preventing cell death, optimizing autophagy, and improving the inflammatory state [164, 165] (Fig. 3). However, intense exercise can also increase inflammation, including generating ROS and reactive nitrogen species (RNS) from damaged muscle, impairing immune function, stimulating inflammation, and draining the glycogen [166, 167]. Compared to vigorous or high-intensity exercise, long-term moderate-intensity exercise tends to generate anti-inflammatory effects, indicating that the effects of exercise on inflammation status largely depend on the duration, intensity, and frequency of exercise [168, 169]. Neopterin is a commonly used marker for assessing levels of inflammation and immune activation, making it a useful tool for monitoring exercise intensity. Three weeks of moderate treadmill exercise (50 min/day, 5 days/week,) can reduce neopterin production and suppress inflammatory responses in mice models with lipopolysaccharide (LPS)-induced inflammation [170]. It is indicated that long-term moderate exercise enhances the immune system and helps prevent infections and chronic diseases [171].

Growing evidence has suggested that appropriate and regular exercise can induce a particular state of anti-inflammation within the body [172]. In addition to enhancing cognitive function by increasing blood flow and oxygen delivery to the brain, regular exercise has been shown to suppress the production of pro-inflammatory markers and their receptors such as IL-1β, IL-6, TNF-α, TNFR1, and TNFR2, and promote the generation of anti-inflammatory molecules like IL-4, IL-1RA, and TGF-β [169, 173] (Table 1). Those molecular markers are recognized as key regulators of the inflammatory response and changes in their production can influence the inflammatory response and the tissue repair process. Compelling evidence has supported the positive role of regular exercise in mediating inflammation status in aging or metabolism diseases. A study has shown that regular moderate exercise significantly decreases circulating levels of IL-6 by 30% and TNF-α by 15% in individuals with metabolic syndromes [174]. Similarly, 16 weeks of aerobic exercise (45–60 min/day, 4 days/week) has been found to alleviate inflammation conditions in chronic inflammatory diseases such as T2DM [175]. In addition, elderly people aged 71 years have been shown to experience a threefold reduction in the number of pro-inflammatory monocytes CD14 and CD16 in their blood after 12 weeks (3 days/week) of endurance (20 min at 70–80% heart rate reserve) and resistance exercise training (two sets of 8RM) [176]. The expression of TLRs on the monocyte membrane is also reduced after prolonged exercise (1.5 h cycling at 75% VO_2max_) [177], which leads to a decrease of co-stimulatory molecules and cytokines of TLR ligands, such as LPS [178]. Together, regular exercise has an overall effect on the immune system and inflammatory responses, which may help alleviate some neuroinflammation-highlighted diseases such as AD.

**Table 1:** The effects of exercise on neuroinflammation in AD patients or animal models

Experimental models/patients	Exercise interventions	Tissues/samples	Intervention effects on inflammatory cytokines	Other outcomes	Studies
Experimental animal models of AD					
Male 3xTg mice, age: 3 months	5 times of 1-h treadmill running per week, 12 weeks	Prefrontal cortex	Reduction of IL-1β, IL-6, and TNFα	i. Decrease of Aβ40 ii. Reduction of microglial and astrocyte activation	Mu et al., 2022 [411]
Male APP/PS1 mice, age: 3 months	5 times of 45-min treadmill running per week, 12 weeks	Hippocampus	Decrease of TNF-α and IL-1β; elevation of IL-10 and TGF-β	i. Enhancement of cognition ii. Elevation of M2 pattern microglia iii. Increase in SOD and reduction of MAD	Zhang et al., 2019 [190]
AD mice, age: 8 weeks	Daily 30 min of treadmill exercise, 1 week	Hippocampus	Reduction of TNF-α and IL-1β	i. Decrease of Aβ ii. Reduction of reactive astrocytes iii. Increase in hippocampal neurogenesis and cognition	Sun et al., 2018 [196]
Male AD rats	5 times of 20 min of treadmill running per week, 4 weeks	Hippocampus	Decrease of TNF-α and IL-1β; elevation of IL-10 and IL-4	i. Decrease of Aβ and p-tau ii. Increment of M2 phenotype microglia iii. Better novel object recognition	Lu et al., 2017 [412]
Male Swiss mice (intracerebroventricular injection with Aβ1 − 40), age: 45–55 days	5 times of 40 min of treadmill exercise per week, 4 weeks	Hippocampus	Reduction of IL-1β, NLRP3 and Caspase-1	Decrease of the number of Iba-1^+^ microglial cells	Rosa et al., 2021 [193]
Tg-NSE/htau23 mice, age: 16 months	5 times weekly of 60-min treadmill exercise, 12 weeks	Hippocampus	Reduction of TNF-α, IL-6, IL-1β, COX-2, and NK-κB	Decrease of GFAP^+^ and MAC-1^+^ glial cells	Leem et al., 2011 [413]
AD mice, age: 17 months	3 weeks of voluntary wheel exercise	Blood	Increase in markers of alternative pathway of the complement system including clusterin, FH and C1INH	Increase in hippocampal neurogenesis and memory function	De Miguel et al., 2021 [21]
Male and female BALB/C mice, age: 4 and 22 months	10 weeks of voluntary wheel exercise	Hippocampus	Reduction of the number of CD86^+^ and MHCII^+^ microglia in female mice; decrease of the number of CD86^+^ microglia but increased MHCII^+^ microglia in male mice	NA	Kohman et al., 2013 [414]
Tg2576 mice, age: 17–18 months	3 weeks of voluntary wheel running	Hippocampus cortex	Reduction of IL-1β and TNF-α; increase in IFN-γ, MIP-1α, MHCII, and CD40	Decrease of Aβ40 and soluble Aβ	Nichol et al., 2008 [415]
Female 5xFAD mice, age: 2–3 months	24 weeks of voluntary wheel running	Blood Hippocampus	Unchanged TNF-α, IL-1β, IL-4, and IL-10	i. Unaltered cognitive function ii. Increase in anxious behavior	Svensson et al., 2020 [416]
Female rats with AD, age: 6–12 months	7 times weekly of 30-min swimming exercise, 4 weeks	Blood	Elevation of IL-10 and reduction of IL-6	i. Improvement of Aβ and tau ii. Increment of BDNF, GSH, and NGF iii. Cognitive enhancement	Medhat et al., 2020 [417]
Male SD rats with AD, age: 2.5 months	Daily 60-min swimming exercise, 4 weeks	Hippocampus	Decrease of TNF-α, IL-1β, IL-6 and IL-18 and increase in IL-10	i. Decrease of Aβ and tau ii. Restoration of learning and memory function	Wu et al., 2018 [310]
Male 3xTg mice, age: 9 months old	3 times weekly of resistance training, 4 weeks	Blood liver frontal cortex hippocampus	Reduction of TNF-α and IL-1β; elevation of IL-10	i. Decrease of Aβ and p-tau ii. Reduction of activation of glia iii. Improvement of memory function	Liu et al., 2020 [418]
Male APP/PS1 mice, age: 6–7 months	5 times weekly of resistance exercise, 4 weeks	Hippocampus	Reduction of IL-1α, IL-4, and IL-6	Decease of Aβ plaques	Hashiguchi et al., 2020 [419]
Human studies					
Female AD patients, *n =* 15, aged 68.3 years	Physical and function training, 2 times weekly of 60-min training, 11 weeks	Blood	Increased levels of IL-4; unchanged IL-10 and TNF-α levels	i. Improvement of cognitive function, spatial judgment and self-awareness ii. Reduction of ROS	de Farias et al., 2021 [420]
AD patients, *n =* 198, aged 71.4–69.9 years	Aerobic exercise, 3 times weekly of 60-min training,16 weeks	Blood CSF	Increase in plasma IL-6 and sTREM2 in CSF	NA	Jensen et al., 2019 [186]
AD patients, *n =* 40, aged 68.9–69.1 years	Treadmill aerobic exercise, 3 times weekly of 45 min of intervention, 8 weeks	Blood	Reduction of plasma TNF-α and IL-6 levels	Improvement of Quality of life	Abd El-Kader and Al-Jiffri, 2016 [421]
MCI patients, *n =* 50, aged 65.7 years	Aerobic exercise, 3 times of 40 min of intervention per week,16 weeks	Blood	Reduction of IL-15 and TNF-α	i. Improvement of cognitive function ii. Elevation of BNDF	Tsai et al., 2019 [422]

AD, Alzheimer's disease; MHCII, major histocompatibility complex II; NA, not applicable; TNF-α, tumor necrosis factor alpha; MIP1-α, macrophage inflammatory protein 1-alpha; Aβ, amyloid beta; TGF-β, transforming growth factor beta; SOD, superoxide dismutase; MAD, methane dicarboxylic aldehyde; NLRP3, NOD-like receptor pyrin domain-containing 3; COX-2, cyclooxygenase-2; NF-κB, nuclear factor kappa B; BDNF, brain-derived neurotrophic factor; GSH, glutathione; NGF, nerve growth factor; sTREM2, soluble triggering receptor expressed on myeloid cells 2; CSF, cerebrospinal fluid; GFAP: glial fibrillary acidic protein; FH, complement factor H; C1INH, complement 1 inhibitor; ROS, reactive oxygen species; min, minute(s); p-tau, phosphorylated tau

### The effects of exercise on neuroinflammation in AD

Physical exercise has positive effects on inflammation in aging and AD humans and animals by decreasing the pro-inflammatory products and enhancing the anti-inflammatory markers [11, 15, 21, 179, 180] (Table 1) (Fig. 3). Exercise can alter the inflammatory pattern in the AD brain and modify the neuropathological properties of the disease [15, 181]. Accordingly, several lines of evidence have shown that physical exercise can enhance the immune system by reducing inflammation and oxidative stress and lead to cognitive improvement by increasing processes of neuroplasticity, such as increasing brain volume and connectivity in older people or those with AD [182–184]. Moreover, other beneficial changes in brain inflammation related to physical exercise are inhibiting complement-related signal pathways in the hippocampus [21] and preserving TREM2 levels in the cerebrospinal fluid (CSF) of AD patients and AD mice model [185–187]. In response to immune insults, microglial cells are stimulated and release pro-inflammatory products to restore cellular balance in AD conditions. However, the persistent activation state and patterns of microglia play a critical role in the neuroinflammation observed in AD [47]. A recent exercise-intervention study has shown that the phagocytic ability of rodent microglial cells has been enhanced tremendously after ten days of treadmill training (25–50 min/day) which leads to the improvement of neuron loss and memory impairment [188]. In addition, 5 weeks of treadmill exercise (5 days/week, 60 min/day) has been found to alleviate hippocampal microglial activation in APP/PS1 rodent models [189]; a 12-week running program (45 min/day, 5 days/week) has been shown to facilitate the transition of microglia from the M1 phase to the M2 phase, resulting in improved neuroinflammation and oxidative stress together with cognitive enhancement in the rodent hippocampus [190]. The interaction between exercise and brain immune responses is thought in part to be mediated by the cell membrane expression of PRRs [191–193], such as TLRs and NLRs. For example, six weeks of treadmill running has been found to have a positive impact on memory performance and reduce brain inflammation in a rodent model of AD, which was involved in decreasing TLR4, nuclear factor kappa B (NF-kB), TNF-α, and IL-1α expression in the rat brain [191]. Moreover, 8 weeks of treadmill exercise (60 min/day, 5 days/week) helps attenuate TLR2-mediated neuroinflammation and cell apoptosis in rodent brains with PD through mediating key markers related to microglial activation (Iba-1), apoptosis (caspase-3 and Bcl-2) and TLR2 downstream signaling cascades including myeloid differentiation factor-88 (MdD88) and tumor necrosis factor receptor-associated factor 6 (TNFR6) [192] (Fig. 3). Compelling evidence from animal experiments has suggested that mediating microglia activation by blocking NLRP3 could be a potential target for preventing AD [194, 195]. Interestingly, a recent study has shown that 4 weeks of treadmill exercise (40 min/day, 5 days/week) can reduce NLRP3 and Caspase-1 expression in the hippocampus of mice that received lateral ventricle injections of Aβ40 [193]. Those findings indicate that targeting microglial PRRs through exercise interventions may have the potential to alleviate neuroinflammation and improve AD pathology.

Exercise-induced improvement of neuroinflammation can lead to an increase in AHN and memory function in the rodent AD model [196] (Fig. 3). Hippocampal neurogenesis impairment often occurs before the onset of AD in adult people and rodent AD models [197, 198], and the aggregation of Aβ has been found to further impair the function of NSCs in the adult hippocampus [199]. Exercise has been shown to generate various beneficial effects on hippocampal neurogenesis. For instance, four months of voluntary wheeling running significantly enhanced hippocampal neurogenesis in the rodent AD model [200]. Moreover, exercise also boosts the generation and maturation of newborn neurons in the adult hippocampus and improves the cognitive function in AD by resorting to BDNF levels [22, 201]. Therefore, it is indicated that exercise can decrease neuroinflammation and increase the number of neurons and glial cells, which provide supportive factors for brain function in AD [15].

BBB dysfunction is a key feature of AD pathology [202, 203]; it affects Aβ clearance, endothelial and pericyte function, tight junction integrity, and microglia activation of the brain [203–205]. Furthermore, when the BBB is damaged, peripheral immune cells and inflammatory cytokines can enter the CNS and cause neuroinflammation [205–207]. By restoring the tight junction proteins in the BBB, exercise can reverse the leakage of the BBB [208] (Fig. 3). Additionally, physical exercise can reduce circulating inflammation (such as reducing IL-1β, IL-6, and TNF-α concentrations) and protect the BBB in T2DM patients [209]. Voluntary running exercise also protects against age-related neurovascular dysfunction, limits the influx of inflammatory products into the CNS, and enhances synaptic plasticity and overall behavioral performance in aged mice [210]. In summary, exercise has the potential to improve neuroinflammation and benefit AD pathology by reducing peripheral inflammation components, affecting cerebral microglial activity, increasing adult neurogenesis, and restoring BBB integrity. Given the close relationship between neuroinflammation and the disease, targeting molecules that regulate the inflammatory response could be a promising therapeutic approach for AD.

## Exercise mimetics: molecular targets for AD

### Exercise mimetics provide a potential therapeutic approach for AD

**Fig. 4:**
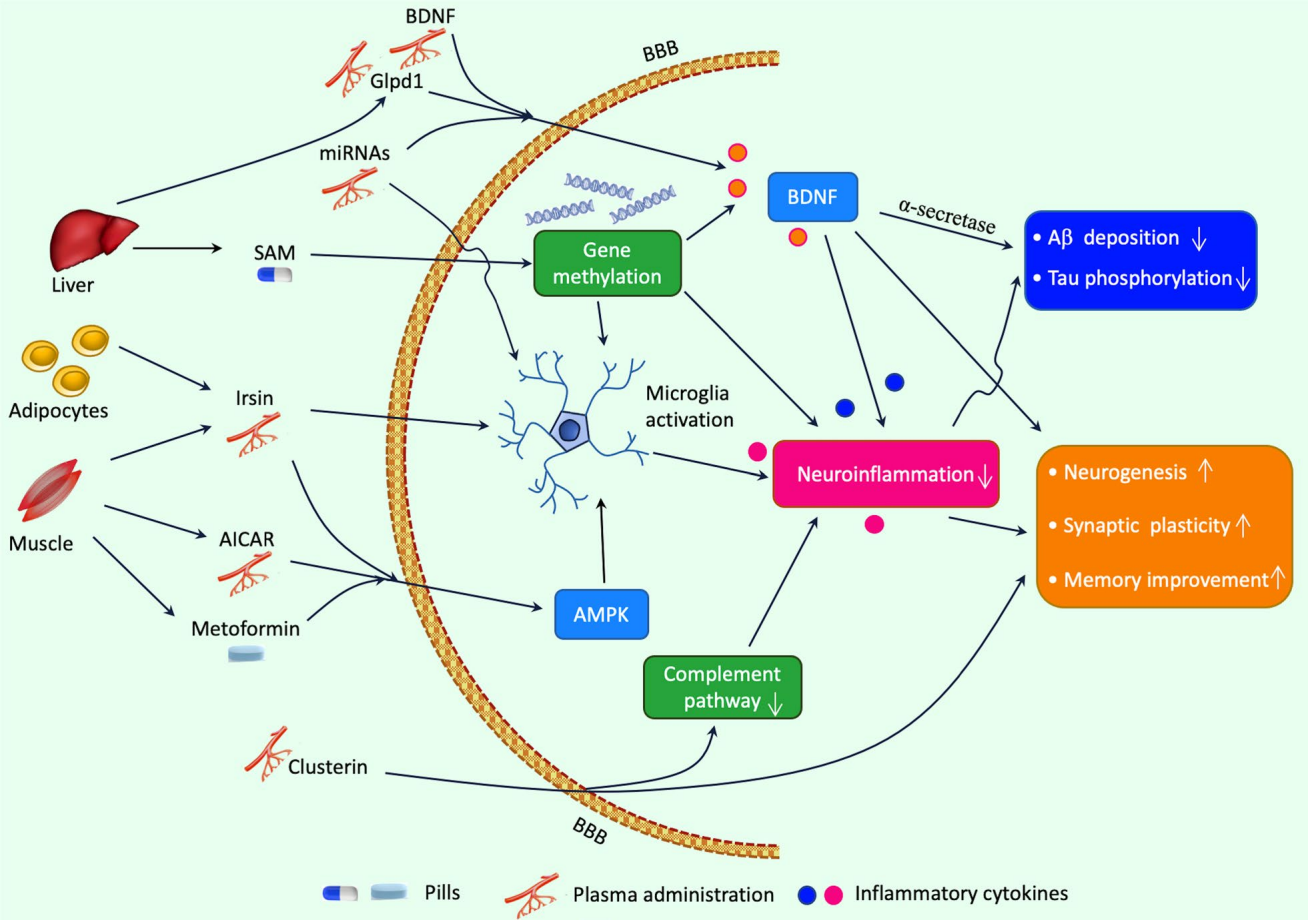
Exercise mimetics for improvement neuroinflammation and AD. The neuroprotective effects of exercise are regulated by a variety of molecular factors that can be activated in a way similar to exercise through the administration of exercise mimetics. These mimetics have been shown to be effective in reducing neuroinflammation and managing AD pathology, making them a valuable alternative for patients who are unable to follow regular physical activity. Exercise benefits the brain through communication between peripheral organs and the brain, such as muscle–brain crosstalk, liver–brain crosstalk, and gut–brain crosstalk. Exercise increases the secretion of FNDC5/irisin from muscles, which can reduce oxidative stress and alleviate neuroinflammation in AD. The liver also generates important factors such as Gpld1 and SAM that are crucial for metabolism and neuroinflammation and can cross the BBB to affect brain function in various AD models. Progress in understanding the molecular mechanisms underlying these interactions endows patients to better utilize the power of exercise mimetics to improve health outcomes. AD, Alzheimer’s disease; BBB, blood–brain barrier; FNDC5, fibronectin type III domain-containing protein 5; Gpld1, glycosylphosphatidylinositol-specific phospholipase D1; SAM, S-adenosylmethionine

Exercise mimetics are a class of molecules that can simulate the beneficial effects of physical exercise on the body and brain [34, 211]. Theoretically, the mimicking impact can involve molecular pathways that directly or indirectly mediate the beneficial effects of exercise. For example, certain exercise mimetics may trigger the same pathways that are activated by exercise, such as AMP-activated protein kinase (AMPK) or PGC-1α [211, 212]. Other exercise mimetics may activate pathways that are downstream or upstream of the exercise-stimulated pathways, such as sirtuins or NF-κB [33, 213]. Exercise mimetics may have considerable potential utility in protecting and treating a wide range of human disorders due to the fact that exercise can generate beneficial effects on diseases involving neurological, psychiatric, cardiovascular, metabolic, inflammatory, and oncological factors [9, 15, 214–218]. Recently, some of these substances have verified their efficacy in preventing AD-related brain damage, such as impaired neurogenesis, synaptic disconnection, memory impairment, and neuropathological alterations [14, 21, 219]. The underlying mechanism by which exercise mimetics prevent AD is not fully understood, but a range of proposed factors are thought to be involved in this effect, such as the reduction in Aβ deposits and NFTs, enhanced neurogenesis and synaptic plasticity, elevated neurotrophin expression, and decreased inflammation and oxidative stress [33, 34, 220]. Exercise has various forms that can induce different effects on individuals by mediating genetics, epigenetics, and various processes at molecular, cellular, and systems levels. We here discuss several promising exercise mimetics, particularly focusing on their potential to restore the therapeutic effects of physical exercise and the underlying mechanisms (Fig. 4). Those molecules or hormones, such as BDNF, FNDC5/irisin, Gpld1, SAM, and microRNA (miRNA), function through facilitating interorgan communications, including muscle–brain crosstalk, liver–brain crosstalk, gut–brain crosstalk, and bone–brain crosstalk. By understanding the molecular mechanisms underlying these interactions, we can better harness the power of exercise mimetics for improved health outcomes.

### BDNF

Exercise has positive effects on the prevention and delay of the onset of AD, and such beneficial effects are believed partly by increasing various neurogenic factors such as the BNDF [23, 221, 222]. BDNF is a key molecule that supports neuronal formation, survival, growth, neurotransmitter regulation, and synaptic plasticity and is engaged in the process of learning, memory, and cognition [223–225]. Given that one pathological feature of AD is synaptic disorder, BDNF has been recommended as a potential predicting biomarker and a therapeutic target for AD [224, 226]. The changed levels and expression of BDNF have been described in the brain and blood of AD patients [226–229], and hypermethylation of the BDNF promoter region, a new approach to evaluate the relationship between BDNF and AD, has been found in postmortem AD brain samples and peripheral blood cells of subjects with AD [230, 231]. The current evidence reveals a close link between BDNF depletion and Aβ deposition, tau hyperphosphorylation, neuroinflammation, and neuronal death [226, 232], which warrants more exploration of its effect and mechanisms in the treatment of AD.

Several studies have demonstrated the beneficial effects of the pharmacological delivery of BDNF in animal models of AD. For instance, BDNF treatment (1-week intravenous injection, 5.7 nmol/kg) in combination with ADTC5 (10 µmol/kg), a molecule facilitating the transport of BDNF across the BBB, induced a marked rise in the cognitive performance and NOR assessments of transgenic AD mice [233]. The treatment also enhanced the number of NG2 glial cells and the expression of EGR1 and ARC mRNA in the brain cortex [233]. Moreover, a three-week running wheel exercise program significantly reduced Aβ levels and increased sAPPα levels in the hippocampus of transgenic AD mice, together with a twofold increment of BDNF levels [24]. Accordingly, BDNF treatment (50 ng/mL) significantly lowered Aβ levels through an α-secretase-dependent mechanism and elevated sAPPα levels in the culture of the human neuroblastoma cell line [24]. Similarly, Parrini and colleagues [234] increased hippocampal BDNF levels in Ts65Dn mice (a transgenic model of Down syndrome) via chronic administration (5 mg/kg body weight, 4 weeks) of 7,8-dihydroxyflavone (DHF). Chronic DHF intervention, in contrast to acute treatment [235], directly induced a 26% increase in phosphorylated tropomyosin receptor kinase B (TrkB) levels and successfully restored hippocampal synaptic plasticity and cognitive function [234]. Recently, one study [219] combined BDNF protein and drug-induced neurogenesis (P7C3) to recapitulate exercise impact on memory performance in a transgenic rodent model of AD. They revealed that the treatment could generate exercise-related neurobiological effects (especially cognitive function), indicating that the usage of exercise mimetics helps promote adult neurogenesis in some brain disorders [219]. Hence, BDNF mimics may provide a therapeutic approach to the treatment of AD by restoring BDNF signaling and mitigating AD pathology.

The ability of BDNF mimics to benefit the cognitive and pathological outcomes of AD may be explained by their interaction with neuroinflammation. One proposed mechanism underlying BDNF-related anti-inflammatory effects is preventing the activation of transcription factors like NF-kB. BDNF can suppress NF-kB activity through several mechanisms, such as binding its receptor TrkB, triggering the PI3K/Akt pathway, or stimulating the SIRT-1 release [236–238]. However, BNDF administration can also activate inflammatory reactions by inducing inflammatory cytokine secretion. Intrathecal injection of BDNF (3 ng/10 µL/rat, every other day for one month) to rats with CYP-induced cystitis enhanced the activation of astrocytes and microglia to release pro-inflammatory cytokines such as TNF-α and IL-1β, exacerbating neuroinflammation and resulting in mechanical allodynia through BDNF-TrkB-p38/JNK signaling [239]. It is suggested that the mediator role of BDNF in neuroinflammation and the subsequent impact on brain disorders such as AD is not fully understood yet. BDNF may have both pro- and anti-inflammatory actions depending on the disease conditions and the molecular pathways triggered [226, 232]. Further research is needed to elucidate the mechanisms of BDNF–neuroinflammation interactions in AD pathology and to identify potential therapeutic strategies to modulate BDNF levels and signaling.

Note that, the delivery of exogenous BDNF is challenging due to its short half-life and poor penetration of the BBB [240, 241]. Fortunately, many strategies have been developed to enhance BDNF levels and signaling endogenously, by directly stimulating its production (for example, increasing endogenous BDNF production drugs and BDNF gene delivery) [226]. Moreover, novel exogenous delivery methods for transporting large molecules, such as the intranasal route and nanoencapsulation technologies, have been extensively studied to overcome the drawbacks of oral or intravenous drug delivery [226, 242–244]. The preclinical data have demonstrated that these particular ways of delivering BDNF can enhance the levels and activity of BDNF and its receptors, leading to improved synaptic communication, adult neurogenesis, and synaptic flexibility [226].

### FNDC5/irisin

Irisin is a newly discovered myokine that has emerged with tremendous interest for its multiple effects on various organs and tissues [245–248]. It is produced from the proteolytic cleavage of a membrane protein FNDC5, which is expressed in both myocytes and brain cells [249–251]. The release of irisin is under the regulation of the transcriptional coactivator peroxisome proliferator-activated receptor gamma coactivator (PGC-1α) during exercise, and muscle and adipose tissue contribute the major sources of circulating irisin levels in rodents [247, 248]. Additionally, the brain can also locally produce irisin and its putative receptor—αVβ5 has been detected in several brain areas, such as the hippocampus, cortex, cerebellum, hypothalamus, and amygdala [28, 249, 252]. Apart from its typical effects on glucose homeostasis, lipid metabolism, insulin sensitivity, and energy expenditure, irisin has been implicated in mediating neuroinflammation, neurogenesis, synaptic plasticity, and cognition in different brain conditions such as AD [16, 246, 253, 254]. Recent clinical studies have provided compelling evidence linking irisin to the development of AD. This evidence includes [255–257] [258, 259]: (i) AD patients often exhibit lower irisin levels in their blood and CSF compared to healthy controls; (ii) irisin levels are inversely associated with AD symptoms, cognitive impairment, brain amyloid deposits, and inflammation; and (iii) specific genetic variations of FNDC5/irisin increase the risk of AD in certain populations. Moreover, the role of irisin as a mediator in AD pathology has been extensively studied in various rodent models of AD using different strategies to alter FNDC5/irisin expression [249, 253, 260–262].

Emerging evidence has indicated that FNDC5/irisin plays a role in mediating the positive impact of exercise on synaptic plasticity, memory, and AD pathology. In a study by Hegazy et al. [263], rats with AD were subjected to 4 weeks of swimming exercise (1 h/day, 5 days/week), which almost rescued the reduced hippocampal FNDC5/irisin expression levels. Notably, this was linked to a decrease in soluble β-amyloid peptide and phosphorylated tau protein levels, improved BDNF and insulin signaling proteins, and ultimately resulted in improved cognitive function [263]. Similarly, a five-week swimming program (1 h per day, 5 days per week) protected against Aβ-induced memory deficits and decreased expression levels of FNDC5/irisin mRNA and protein in mouse hippocampi [16]. However, intraperitoneal administration of an anti-FNDC5 agent also blocked the protective effects of exercise against impairments in synaptic plasticity and memory caused by Aβ [16]. The results indicate that FNDC5/irisin is crucial in mediating the neuroprotective benefits of exercise on synaptic plasticity and memory in AD. This suggests that administering irisin may provide a therapeutic avenue for AD pathology by conferring the beneficial effects of exercise. For example, Bretland et al., [264] showed that a 4-week injection of recombinant (r)-irisin (100 µg/kg weekly) significantly decreased the tau phosphorylation load and inflammatory cytokine levels such as TNF-α in the hippocampus of a transgenic rodent model of AD. Similarly, the r-irisin administration also protested against Aβ-related impairment in NOR and fear conditioning memory in murine AD models [16]. Interestingly, blocking of FNDC5/irisin activation with an antibody could inhibit the exercise-induced improvement of synaptic plasticity and memory function in AD mice [16].

The beneficial effects of irisin administration on AD pathology and cognitive function are proposed to be due, in part, to the modulation of the modulating anti-inflammatory response in the CNS. Irisin treatment markedly reduces the expression of genes specific to astrocytes and microglia, and peripherally increasing irisin levels improves cognitive function in AD mice by interacting with αVβ5 integrin receptor complexes [254]. Moreover, irisin intervention can also enhance spatial learning and memory, reduce neuronal apoptosis, and modulate the inflammatory response by inhibiting microglia/macrophage activation, neutrophil infiltration, IL-1β expression, and promoting the phenotypic switch of microglia/macrophage in a murine model of the brain injury [249]. Additionally, irisin treatment shows therapeutic potential for cognitive impairment and synaptic plasticity by modulating neuroinflammation, astrocyte activation, and the expression of P38, STAT3, and NFκB signaling molecules in the rodent diabetic brain [253]. Several pathways have been proposed to explain the anti-inflammatory and anti-neurotoxicity effects of irisin in CNS. One pathway involves the binding of irisin to integrin αVβ5, a receptor expressed in glial cells in the CNS [249, 265]. This binding activates AMPK, an enzyme that regulates cellular metabolism and inflammation, which then inhibits NF-κB signaling that promotes pro-inflammatory gene expression [16, 262]. Another pathway requires the participation of BDNF. BDNF is a neurotrophic factor that has anti-inflammatory and neuroprotective effects on the CNS [259, 266]. Irisin stimulates BDNF production in hippocampal neurons and astrocytes through PGC-1α/FNDC5 signaling [19]. BDNF then activates its receptor TrkB and downstream signaling pathways such as Akt/CREB, and Erk1/2/MAPK, which modulate synaptic plasticity, neuronal survival, and inflammation [259]. Taken together, irisin treatment might be a potential strategy for alleviating neuroinflammation and AD symptoms, perhaps by modulating cytokine production, glial activation, hippocampal synaptic plasticity, Aβ formation and accumulation, tau abnormal phosphorylation, and excessive oxidative stress [253, 254, 258]. However, to be a feasible strategy, there are still some key questions that need to be addressed. These include determining the optimal dose, route, frequency, and duration of irisin administration for different stages and subtypes of AD. Validating the safety and efficacy of irisin in humans through clinical trials and monitoring potential side effects or adverse reactions is also necessary. Finally, improving the stability and bioavailability of irisin through novel formulations or delivery systems is crucial.

### Clusterin

CLU, also referred to as apolipoprotein J (ApoJ), is a protein that is highly expressed in the brain, especially glial cells and neurons where it mediates cholesterol metabolism, oxidative stress, and cell apoptosis [267–269]. In the context of AD, CLU has been implicated in the Aβ formation and mediates its clumping and clearance [270–272]. Growing evidence has revealed that CLU levels are increased in AD-affected brain regions [273, 274] and in the CSF of AD patients [275]. For instance, Thambisetty et al. [276] reported that CSF levels of CLU were significantly higher in AD patients compared with controls, and Schrijvers et al. [277] revealed that higher plasma CLU was associated with disease prevalence and severity of AD. Compelling evidence has suggested that there is a close link between the levels of CLU and the occurrence, development, and severity of AD [277–279], indicating that CLU may serve as a peripheral indicator of AD. Although the precise function of CLU in AD pathogenesis is not fully understood, there is evidence that it plays a part in removing the Aβ peptides [280] and mediating the tau phosphorylation [281, 282]. For example, a study by DeMattos et al. [280] reveals that CLU binds to Aβ peptides and facilitates their clearance from the brain in the rodent model of AD.

Exercise is known for its potential benefits on AD onset and the development of disease pathology [13, 15, 283]. Emerging evidence has indicated this positive effect is linked to its effect on CLU expression. A 14-day treadmill training significantly elevated adipocytic CLU levels, which inhibited the cortical complement pathway and prevented motor dysfunction worsening in a rodent model of overexpressing mutant human TDP-43 [284]. Moreover, serum levels of CLU were markedly elevated after 6-month exercise intervention (30 min/day, 3 days/week, intensity at 60–70% of maximum heart rate) in the patients with amnestic mild cognitive impairment compared to basic values before interventions [21]. It is indicated that CLU could be an "exercise factor" that benefits brain function in neurological disorders. One recent study collected “runner plasma” from exercised mice and infused it into sedentary mice leading to a markedly increased number of neural stem/progenitor cells and DCX^+^ neuroblasts, enhanced contextual learning and memory function, and decreased expressions of inflammatory genes in young rodent hippocampus [21]. Runner plasma decreased transcripts related to neuroinflammation and suppressed inflammatory reactions. Subsequent analysis confirmed an increment in complement cascade inhibitors-CLU [21], which is responsible for the exercise-related anti-neuroinflammatory reactions in AD mice [285]. It is suggested that CLU acts as a pivotal molecule in regulating the beneficial effects of exercise on brain plasticity and function, and various therapeutic methods have aimed to target CLU pharmacologically to reduce AD pathology. A recent in vivo study has revealed that CLU-deficient mice exhibit impairment in excitatory synaptic transmission and spine density, which can be restored by increasing CLU secretion from astrocytes; additionally, increasing CLU expression can increase excitatory neurotransmission, reverse synaptic damage, and alleviate AD pathology in transgenic AD mice [267]. Apolipoprotein E (ApoE) is known to mediate the inflammatory response in AD pathology [286, 287]. Oral ApoJ peptides (D-[113–122]apoJ, 125 or 250 µg/mouse/d) can improve HDL inflammatory characteristics in mice and monkeys, as well as reduce lesion formation in ApoE-deficient mice [288]. Additionally, treatment of CLU at a concentration of 10 nM significantly increases neuronal differentiation from human NPCs and reduces cell apoptosis [289]. The effectiveness of exogenous CLU treatment depends partly on its ability to cross the BBB, which can be facilitated by several mechanisms, including receptor-mediated transport via gp330/megalin receptors in the choroid plexuses and vascular epithelium and the formation of complexes with other molecules such as Low-density lipoprotein [290]. However, while targeting CLU may provide new therapeutic opportunities for AD prevention or treatment, there are challenges associated with this approach. The role of CLU in AD is complex and context-dependent, as it can act as either a protective or harmful factor depending on its interaction with other molecules or cellular processes [270, 272, 290]. Therefore, further research is needed to fully understand the molecular mechanisms and regulation of CLU in AD, which may provide new insights into the etiology, progression, and clinical implications of this devastating disease.

### Gpld1

Recently, several novel circulating factors have emerged with great interest for their evident bioactive role in mediating the communication between peripheral organs and the brain during exercise [291, 292]. Among the various biomarkers, Gpld1 represents a potent and promising exercise mediator that functions as an enzyme to cleave GPI molecules from the cell membrane, thus playing a crucial role in regulating various cellular processes [22, 292]. Gpld1 is predominantly synthesized by the liver and circulates in the blood affecting diverse metabolic and inflammatory processes [29, 293–296]. The expression of Gpld1 has been detected in the brain [297, 298], modified by exercise [22, 299], and involved in neuronal plasticity and cognitive functions in health and diseases [29, 48, 300]. It is indicated that Gpld1 mimic may have the potential to restore brain plasticity and function in neurological disorders. Recently, Horowitz et al. [22] conducted a voluntary wheel-running program on aging mice (18 months) and revealed that hippocampal neurogenesis and memory performance were markedly enhanced. Interestingly, the “runner plasma” isolated from exercising old mice was intravenously transferred (eight times for 3 weeks) to sedentary old mice, resulting in a significant increase of hippocampal neurogenesis, BDNF up-regulation, and learning and memory improvement in aged mice [22]. To identify which factor accounts for exercise-induced effects, they analyzed the runner plasma and proposed Gpld1 as the candidate mediator. Moreover, in a subsequent exercise-intervention study (6-week voluntary wheel running), circulating Gpld1 levels were higher in active healthy elders compared to sedentary individuals, and the major source of Gpld1 was derived from the liver [22]. To mimic exercise effects, they increased hepatic Gpld1 expression to raise circulating GPI levels which was enough to improve hippocampal neurogenesis and cognitive performance in aged rodent hippocampus. To further verify the crucial role of Gpld1, they overexpressed a mutated form of Gpld1 that effectively blocked Gpld1 function and decreased its beneficial effects on neurogenesis and cognitive functions [22]. It is proposed that the favorable effects of exercise on the CNS can be conferred by the administration of exercise mimetics such as Gpld1 through liver–brain crosstalk, suggesting that Gpld1 has the potential to restore neuronal plasticity and function in brain disorders such as AD [22, 292].

### SAM

SAM is referred to as a type of methyl donor, a molecule that facilitates the transfer of a methyl group to another molecule via the process of methylation [301, 302]. In recent years, methyl donors have gained immense popularity for their essential role in various biological processes such as DNA methylation (DNAm). DNAm can modulate gene expression, protein production, neurogenesis, and neurotransmitter metabolism [303]. These processes are often disrupted in neurological disorders such as AD [304, 305]. Emerging evidence has indicated changes in methylation profiles of specific genes or regions in the brain of AD patients, suggesting that methylation may be involved in the pathogenesis of AD. A recent study conducted a meta-analysis of methylation data in different brain regions of individuals with AD [305]. The study revealed that methylation changes related to AD were predominantly enriched in genes that play a crucial role in neurodevelopment and neurogenesis [305]. Additionally, Altuna et al. [304] particularly focused on genome-wide DNA methylation levels in the hippocampus of AD individuals. They discovered 118 differentially methylated positions (DMPs) related to AD and these DMPs were remarkably associated with the phosphorylated tau protein burden [304]. Subsequent functional analysis revealed that these DMPs were enriched for genes involved in neural development and hippocampal neurogenesis that were altered in the progression of AD pathology [304]. The actions of DNA methylation are dynamically regulated by diverse factors including DNA repair, oxidative stress, inflammation, environmental stimuli, and exercise [306]. These modifications in DNAm can subsequently impact brain functions such as neurogenesis, synaptic plasticity, and cognition, indicating a possible therapeutic implication of DNAm in regulating the development and progression of AD pathology [306].

Exercise is a dynamic mediator for methylation patterns in brain health and disorders [307]. For example, in a group of older African Americans with mild cognitive impairment, a 6-month program of aerobic exercise (40 min/day, 3 days/week) caused alterations in global DNA methylation patterns [308]. These changes affected genes such as VSP52, SACRB1, ARTN, NR1H2, and PPPLR5D, which are involved in various processes including amyloid formation, intracellular protein transport, and lipoprotein mediation [308]. Exercise also modifies the activity of the enzyme DNMTs, which are involved in the regulation of neuronal survival and the methylation processes in aging- and disease-associated neurodegradation [309]. Swimming exercise (1 h/day, 26 days) results in a significant increase in the protein levels and DNA-binding activity of nuclear factor erythroid 2-related factor 2 (Nrf2) in a rodent model of AD, which leads to subsequent inhibition of the expression of downstream antioxidant genes [310]. Given the significant role of methylation of genes in the beneficial effects of exercise on brain function, it may be possible to develop a potential therapeutic approach for brain disorders like AD by targeting DNAm and related signaling pathways, such as methyl donors.

As an essential methyl donor, SAM is primarily synthesized in the liver and plays an essential role in various biological processes affecting signaling pathways involved in cell growth, apoptosis, and immune function [311–313]. SAM could be utilized as both a dietary supplement and a prescription drug for a range of conditions, such as depression, anxiety, liver disease, osteoarthritis, and AD. SAM administration can help ameliorate AD pathology and improve cognitive function. A study conducted on 3xTg-AD mice showed that SAM administration (100 mg SAM/kg) decreased hippocampal intracellular Aβ deposits and phosphorylated tau immunoreactivity [314]. SAM therapy showed a temporal pattern in altering AD neuropathology, reducing extracellular Aβ deposition by 80% in 11-month-old mice after a 1-month treatment but only by 24% in 15.5-month-old mice after a 3-month intervention [314]. The anti-AD effects are believed to be regulated in part by the ability of SAM to normalize gene methylation. Fuso et al. [315] found that SAM treatment can restore normal gene expression, reduce presenilin1 expression, and decrease Aβ levels in conditions associated with AD onset. Additionally, SAM may impact Aβ metabolism by regulating its formation, clearance, and aggregation. As a methyl donor, SAM affects the activity of enzymes, such as secretases, involved in the synthesis or cleavage of APP [316]. SAM can also enhance Aβ clearance by increasing the expression of ApoE [317] and prevent Aβ aggregation by disrupting its interaction with metal ions or altering its conformation [318].

SAM therapy can benefit AD by modulating some of the pathological processes such as oxidative damage, inflammation, mitochondrial impairment, and cholinergic deficiency. For example, SAM has antioxidant effects by targeting glutathione (GSH) and its transferase, which are important for quenching oxidative species and eliminating toxic xenobiotics. SAM supplementation restored glutathione S-transferase activity and eliminated reactive oxygen species in transgenic ApoE^−/−^ mice, highlighting its critical role in maintaining neuronal health, particularly in AD [319, 320]. Moreover, administration of SAM (16 mg/kg for 4 weeks) reverses cognitive impairment in D-galactose-induced brain aging rats and rescues neuron loss, elevates BDNF levels in the hippocampus, decreases amyloid-β deposits, and inhibits microglia activation and pro-inflammatory cytokine levels in the hippocampus and serum [321]. Additionally, SAM injection (10 mg/kg daily for 6 weeks) could protect brain cells against Aβ-induced cellular injury by inhibiting neuroinflammation and oxidative stress [322]. Mitochondrial dysfunction is a hallmark of brain disorders such as AD [323], which could be a potential target for treatment. Recently, Lam et al. [324] reported that dietary vitamin B12 can alleviate mitochondrial fragmentation, bioenergetic defects, and oxidative stress, delaying Aβ-induced paralysis in a methionine/SAM-dependent manner. Together, SAM administration has shown multiple favorable effects on AD by modifying various pathological processes involved in the disease [325]. However, SAM therapy may face some challenges or limitations such as the lack of a well-established optimal dose, duration, and route of administration for AD, as well as the possible side effects. Therefore, further research is necessary to address those concerns.

### MicroRNAs

MiRNAs are a class of short (18–25 nucleotides) non-coding RNAs that bind to the 3' untranslated region of target mRNAs affecting their translation or stability [326, 327]. MiRNAs can affect various aspects of immune responses by modulating the expression of key molecules involved in inflammatory signaling pathways, such as NF-κB, MAPK, and TLRs [328, 329]. MiRNAs can have either pro- or anti-inflammatory effects depending on their target genes and the cellular context [328–330]. Given the essential roles of miRNAs in immune regulation and AD pathology [331, 332], they have emerged as promising biomarkers and therapeutic targets for AD [333]. In addition to being critical molecules in modulating neuroinflammation and AD pathology [331, 332], miRNAs also interact with lifestyle factors such as physical activities. For example, both acute and chronic exercise can markedly affect miRNA expressions in various tissues and fluids [334–336], such as skeletal muscle, brain, blood, and CSF. These miRNAs are involved in various biological processes [337, 338], including cell differentiation, proliferation, apoptosis, metabolism, and immune response, as well as pathological processes of various diseases, such as diabetes, heart diseases, cancer, neurodegenerative disorders, neuroinflammation, and mental disorders [337, 338]. Physical exercise is recognized as a non-pharmacological regimen for improving memory function and delaying the onset and progression of AD [15, 283]. Therapeutic strategies that target miRNAs and relevant signaling molecules involved in exercise effects have been proposed to ameliorate neuroinflammation and AD pathology [333, 339, 340]. Those exercise mimetics can be recombinant oligonucleotides, such as miRNA mimics, antagomirs, and locked nucleic acids, or viral vectors that deliver miRNA genes or miRNA inhibitors into particular brain areas or cell types [340, 341].

MiR-132 is one of the most markedly changed miRNAs in AD brains and CSF and is also the most widely investigated one due to its essential role in mediating some genes critical for synaptic transmission, neurogenesis, neuroinflammation, and tau phosphorylation [340]. The expression of miR-132 is down-regulated by Aβ pathology in the hippocampus of transgenic AD mice, whereas one-month voluntary running upregulates miR-132 expression and improves cognitive function [342]. However, intracerebroventricular delivery of anti-miR-132 oligonucleotide can abolish the exercise-induced favorable effects on hippocampal neurogenesis and cognition as well as BDNF expression, indicating that miR-132 can be a therapeutic target [342]. Interestingly, restoring down-regulated miR-132 levels by delivery of its mimic in the hippocampus of AD mice rescues AHN and memory impairment [342]. Similarly, data from other experiments also verified that delivering miR-132 synthetic agents into the brains of diverse rodent AD models (such as APP/PS1 and 3xTg AD mice) could alleviate the accumulation of Aβ 40–42 and Tau hyperphosphorylation and restore hippocampal neurogenesis and cognitive function [343, 344]. Those results suggest that targeting miR-132 signaling may have therapeutic potential for the treatment of AD.

MiR-146 is among the commonly dysregulated miRNAs with known or potential neuroimmune roles in AD [345]. MiR-146 is highly expressed in microglia and inhibits the NF-κB signaling pathway by directly affecting IL-1 receptor-associated kinase 1 and TNF receptor-associated factor 6 [346, 347]. MiR-146 depletion mice do not show effective microglia-regulated phagocytosis in response to LPS, indicating that miR-146 is crucial for the microglial reaction to inflammation [348]. Exercise can alter the expression and function of miR-146 in various tissues [349, 350], indicating that the changed miR-146 profiles may modulate the positive effect of exercise on health and diseases. To test the treatment effects of miR-146a on AD pathology, Mai et al. intranasally delivered a miR-146a agomir (M146AG) to transgenic AD mice and they revealed that the treatment could alleviate amyloid and Tau pathologies and improve neuroinflammation and cognitive function [351].

Recently, increasing studies have shown interest in the therapeutic potential of targeting miRNAs for treating or delaying the onset and progress of AD. For example, some studies revealed that AD patients had elevated miR-155 levels in blood-derived monocytes and monocyte-derived macrophages [352], as well as in the neocortical extracellular fluid and CSF [353]. Moreover, the enhanced production of miR-155 was associated with upregulated activation of glial cells and downregulated SOCS-1 in the murine model of 3xTg AD [354]. Those data indicate that miR-155 could be a potential therapeutic target. Accordingly, reducing miR-155 levels by administrating curcumin efficiently improved the activation of microglia cells and related neurodegenerative phenotype in ApoE3-5xFAD mice [355]. Similarly, the delivery of anti-miR-155 agents reduced the expression of pro-inflammatory cytokines (such as IL-1β, IL-6, and TNF-α) and Caspase-3, leading to the improvement of impaired learning functions [356]. Additionally, a number of miRNAs, such as miR-7, miR-21, miR-339, and miR-29, have been identified as exercise mediators and have shown therapeutic potential in treating AD [11, 357–359]. Taken together, a variety of miRNAs have been found dysregulated in AD conditions, indicating a crucial role in neuroinflammation and AD. Their therapeutic potential has been investigated and some of the agents have shown efficacy. However, some problems emerge when using miRNAs as a therapeutic approach, for example, some studies reveal that the administration dose has to be carefully controlled, as memory performance can be adversely affected by miR-132 levels that are more than threefold higher than normal [342, 343]. This suggests that there are still some challenges and limitations that need to be addressed in the development and application of miRNA-targeted strategies for AD, especially the specificity, stability, efficiency, and safety of miRNA agents.

### 5-Aminoimidazole-4-carboxamide ribonucleotide (AICAR)

AMPK is recognized as a key mediator of cellular metabolism and it triggers a plethora of cellular and molecular factors to meet metabolic demands during exercise [360–362]. Additionally, AMPK activation has been shown to have anti-inflammatory effects in different cell types, such as macrophages, glial cells, neuronal cells, myocytes, adipocytes, and hepatocytes [360, 363, 364]. AMPK activation suppresses the NF-κB signaling pathway in response to hypoxia and reoxygenation [365], a crucial regulator of the inflammatory transcript expression [366]. AMPK inhibits NF-κB signaling by phosphorylating and suppressing the inhibitor of κB kinase (IKK), which blocks the phosphorylation and degradation of the inhibitor of κB (IκB) and thus prevents the nuclear translocation and activation of NF-κB [213]. AMPK also induces phosphorylation and inhibition of the p65 subunit of NF-κB through sirtuin (SIRT)1, a deacetylase that interacts with AMPK [367, 368]. By inhibiting NF-κB signaling, AMPK reduces the expression of pro-inflammatory cytokines (such as TNF-α, IL-1β, IL-6) and chemokines (such as CXCL1) in various cell types and tissues. In addition to inhibiting NF-κB signaling, AMPK activation also modulates other signaling pathways that are involved in neuroinflammation, such as the mammalian target of rapamycin, NLRP3 inflammasome, Janus kinase/signal transducer and activator of transcription, MAPK, and Nrf2, leading to suppression of protein synthesis and cell growth, reduction of IL-1β and IL-18, increment of anti-inflammatory gene expression, and improvement of antioxidant defense and oxidative stress [369, 370].

Exercise has beneficial effects on brain disorders by enhancing neurogenesis, synaptic plasticity, and cognitive performance [371–373]. It is well-documented that AMPK is a pivotal mediator of exercise-induced beneficial effects on neuroinflammation and neurodegeneration in animal models of neurological disorders [374]. For example, exercise reduced microglial activation and neuroinflammation in a mouse model of AD [375, 376]. Exercise also attenuated neuroinflammation and neuronal death in rat models of ischemic stroke [377, 378] and PD [371, 379]. These findings suggest that exercise-activated AMPK signaling is a molecular target for modulating neuroinflammation and improving brain health in various disorders.

Several compounds targeting AMPK signaling have been developed and verified as effective in animal models of neurological disorders. One such compound is AICAR, an analog of AMP and the product of the purine synthesis pathway [213]. The phosphorylation of AICA-riboside is under the regulation of the cellular adenosine kinase, which activates AMPK by binding its γ subunit and inducing its autophosphorylation Thr172 [380]. AICAR can influence various organs and tissues and modulate different physiological and pathological processes [381, 382], in particular, modulating inflammation by directly activating AMPK and lowering the production of inflammatory cytokines [383, 384]. For example, AICAR treatment reduced the inflammation and cytokine production in human aortic smooth muscle cells in a dose-dependent manner [385]. Administration of AICAR promoted the expression of insulin-degrading enzyme (IDE) and reduced Aβ deposition in mice with AD, resulting in enhanced spatial learning and recognition performance [386]. Moreover, Du et al. revealed that all AD-like pathological changes including biochemistry and cognitive function could be alleviated in AICAR-treated rats [387]. AICAR treatment has inhibitory effects on LPS/Aβ-induced inflammatory response by reducing the production of pro-inflammatory cytokine (such as TNF-α, IL-1β, and IL-6) and by attenuating ROS generation and glutathione depletion in glial cells [384]. It is implicated that the beneficial impact of AICAR on AD conditions may be correlated to its anti-inflammatory effects. However, the anti-inflammatory action of AICAR may depend on the treatment duration. One study reported that the administration of AICAR (500 mg/kg, for 3, 7, and 14 days) had a similar effect to exercise on muscle AMPK signaling in young male mice, which in turn increased the number of new neurons and BDNF levels in the dentate gyrus [388]. However, the compound did not enhance DG cell proliferation and neurotrophin levels after 14 days of treatment but elevated apoptotic gene expression and inflammatory cytokine production (such as IL-1β) [388]. The findings are consistent with a previous study that reported no improvement in spatial memory and adult neurogenesis after longer (14 days) AICAR administration, suggesting that the effects of AICAR on the brain depend on the duration of its administration and involve divergent underlying mechanisms [389].

### Metformin

Metformin is another compound that can activate AMPK signaling, which is a first-line antidiabetic drug with insulin-sensitizing effects and can cross the BBB. Metformin has pleiotropic effects on various cellular processes, including energy metabolism, oxidative stress, inflammation, mitochondrial function, and adult neurogenesis [390–392]. These effects may implicate its mediator role in AD prevention and treatment due to its capacity to modulate some key mechanisms involved in AD pathogenesis such as reduction of neuroinflammation, clearance of A, modulation of tau phosphorylation, and induction of neurogenesis [61, 393, 394]. Similar to exercise, metformin is a common treatment for patients with T2DM, which is effective in lowering blood glucose levels by enhancing the expression and membrane translocation of glucose transporter-4 in adipose tissue and skeletal muscle, thereby promoting glucose uptake [395, 396]. Moreover, metformin treatment also inhibits TNF-α-regulated inflammatory processes in vascular smooth muscle cells [397]. Metformin has been shown to suppress IL-1β expression in macrophages during chronic exposure to LPS by reducing ROS levels through activating AMPK pathways [398]. Additionally, metformin treatment also reduced the transcript expression of inflammatory markers such as TGF-β, ICAM-1, and NF-kB cultured murine glomerular mesangial cells [399]. Current evidence indicates that metformin may have anti-inflammatory effects similar to exercise in part by suppressing the production of inflammatory cytokines such as TNF-α, TLR2/4, and IL-6 [400, 401].

The relationship between metformin use and the risk of AD has been extensively investigated, but the findings are not consistent or conclusive. Some studies have shown that metformin use may reduce the risk of AD and improve cognitive function in diabetic patients [402, 403]. Using a Taiwanese national health insurance database, Hsu et al. [404] matched 800 T2DM patients who developed dementia with 3200 controls. They reported that metformin use reduced the risk of dementia (adjusted odds ratio [AOR] 0.46; 95% CI 0.35–0.61), especially AD (AOR 0.38; 95% CI 0.25–0.58), compared with controls without metformin use. Another study [405] used data from a prospective cohort data of elderly Latinos in the US to examine the effect of metformin on cognition. They used a battery of neuropsychological tests to measure cognitive function and found that participants with diabetes without metformin use had worse cognitive scores than those without diabetes or metformin use, while participants with diabetes with metformin use had comparable cognitive scores to those without diabetes. Contrarily, using a UK primary care database, Imfeld et al. [406] matched 17,415 diabetes patients who developed dementia with 68,875 controls. They reported that metformin use increased the risk of dementia (AOR 1.23, 95% CI 1.13–1.34), especially AD (AOR 1.29, 95% CI 1.15–1.44), compared with non-use of metformin. Another population-based study using a Korean national health insurance database matched 1675 newly diagnosed T2DM patients who developed AD with 8375 controls [407]. They revealed that metformin use increased the odds of AD (AOR 1.50, 95% CI 1.23–1.83), and this effect was stronger in patients with longer diabetes duration (> 10 years) and higher cumulative doses (> 1000 g) of metformin. However, those studies also had some limitations, such as insufficient data on lifestyle factors, potential misdiagnosis of AD, and possible remaining confounding by indication.

One recent meta-analysis combining data from 19 studies revealed that metformin had no significant impact on improving cognitive function or protecting against dementia risk [408]. However, some researchers pointed out that the associations between metformin use and AD risk or cognition may be influenced by some factors, such as diabetic status, Apoe-ε4 status, vitamin B12 deficiency, and homocysteine levels [402, 409]. For instance, Ng et al. [410] performed a meta-analysis of observational studies and reported that metformin use reduced the risk of AD in patients with diabetes (risk ratio [RR] 0.76, 95% CI 0.63–0.92), but not in patients without diabetes (1.05, 95% CI 0.76–1.46). The underlying reasons for the discrepancy across studies may include variations in study design, population characteristics, confounding factors, and outcome measures. Together, since metformin use has been shown to reduce Aβ deposition and abnormal tau phosphorylation, alleviate inflammation, moderate insulin-sensitizing, and enhance the neurogenesis [393, 394], all of which are key mechanisms for AD, the relationship between metformin use and AD risk warrants further investigation.

## Perspective

As our society continues to age, the prevalence of neurological diseases and healthcare costs are increasing, which require effective strategies to address these issues. An active lifestyle is a preferred choice for enhancing brain health, and, for those who are immobile due to diseases, injuries, or frailty, factors derived from runner plasma can be a valuable alternative. Though we do not fully comprehend the exact mechanism of how these molecules work, they have been proven to be an effective regimen for some neurodegenerative disorders such as AD. These molecules have been shown to improve cognitive function, reduce amyloid accumulation, and increase neurogenesis and synaptic plasticity in animal models and human studies of AD. Exercise mimetics may also have an impact on neuroinflammatory responses and autophagy, which contribute to the accumulation of toxic aggregates and neuronal dysfunction of AD.

This review discussed recent evidence regarding certain molecules that partially exhibit beneficial effects similar to exercise on neuroinflammation and AD. However, this concept is still in its early stages, facing many challenges that need to be addressed. First, it is crucial to note that the holistic and multifaceted impact of exercise on the CNS is unlikely to be replicated by delivering a single factor. Moreover, the benefits of exercise in the context of AD are likely related to several molecules, with many potentially still unknown. The molecules that have been tested represent only a small fraction of the molecules involved in exercise-related effects on AD. This underscores the necessity for future studies to explore molecules that mimic the benefits of exercise comprehensively. Additionally, while animal studies provide evidence of the beneficial effects of exercise mimetics on AD, clinical data among AD patients are limited. Therefore, it is necessary to establish the safety, dosage, duration, and combination of these molecules for AD patients. Together, it is crucial to address those issues about exercise mimetics, and future studies are needed to confirm their effectiveness and safety in humans.

## Data Availability

Not applicable.

## References

[1] Atella V, Piano Mortari A, Kopinska J, Belotti F, Lapi F, Cricelli C, Fontana L (2019). Trends in age-related disease burden and healthcare utilization. Aging Cell.

[2] Hou Y, Dan X, Babbar M, Wei Y, Hasselbalch SG, Croteau DL, Bohr VA (2019). Ageing as a risk factor for neurodegenerative disease. Nat Rev Neurol.

[3] Scheltens P, De Strooper B, Kivipelto M, Holstege H, Chetelat G, Teunissen CE, Cummings J, van der Flier WM (2021). Alzheimer’s disease. Lancet.

[4] Rajan KB, Weuve J, Barnes LL, McAninch EA, Wilson RS, Evans DA (2021). Population estimate of people with clinical Alzheimer’s disease and mild cognitive impairment in the United States (2020–2060). Alzheimers Dement.

[5] Joe E, Ringman JM (2019). Cognitive symptoms of Alzheimer’s disease: clinical management and prevention. BMJ.

[6] McGee SL, Hargreaves M (2020). Exercise adaptations: molecular mechanisms and potential targets for therapeutic benefit. Nat Rev Endocrinol.

[7] Kelly RS, Kelly MP, Kelly P (2020). Metabolomics, physical activity, exercise and health: a review of the current evidence. Biochim Biophys Acta Mol Basis Dis.

[8] Cotman CW, Berchtold NC, Christie LA (2007). Exercise builds brain health: key roles of growth factor cascades and inflammation. Trends Neurosci.

[9] Alkadhi KA (2018). Exercise as a positive modulator of brain function. Mol Neurobiol.

[10] Giorgetti E, Panesar M, Zhang Y, Joller S, Ronco M, Obrecht M, Lambert C, Accart N, Beckmann N, Doelemeyer A (2019). Modulation of microglia by voluntary exercise or CSF1R inhibition prevents age-related loss of functional motor units. Cell Rep.

[11] Li Z, Chen Q, Liu J, Du Y (2020). Physical exercise ameliorates the cognitive function and attenuates the neuroinflammation of Alzheimer’s disease via miR-129–5p. Dement Geriatr Cogn Disord.

[12] McDonnell MN, Smith AE, Mackintosh SF (2011). Aerobic exercise to improve cognitive function in adults with neurological disorders: a systematic review. Arch Phys Med Rehabil.

[13] Adlard PA, Perreau VM, Pop V, Cotman CW (2005). Voluntary exercise decreases amyloid load in a transgenic model of Alzheimer’s disease. J Neurosci.

[14] Baranowski BJ, Mohammad A, Finch MS, Brown A, Dhaliwal R, Marko DM, LeBlanc PJ, McCormick CM, Fajardo VA, MacPherson REK (2023). Exercise training and BDNF injections alter APP processing enzymes and improve cognition. J Appl Physiol (1985).

[15] Wang M, Zhang H, Liang J, Huang J, Chen N (2023). Exercise suppresses neuroinflammation for alleviating Alzheimer’s disease. J Neuroinflammation.

[16] Lourenco MV, Frozza RL, de Freitas GB, Zhang H, Kincheski GC, Ribeiro FC, Goncalves RA, Clarke JR, Beckman D, Staniszewski A (2019). Exercise-linked FNDC5/irisin rescues synaptic plasticity and memory defects in Alzheimer’s models. Nat Med.

[17] Reddy I, Yadav Y, Dey CS (2023). Cellular and molecular regulation of exercise-a neuronal perspective. Cell Mol Neurobiol.

[18] Lu Y, Bu FQ, Wang F, Liu L, Zhang S, Wang G, Hu XY (2023). Recent advances on the molecular mechanisms of exercise-induced improvements of cognitive dysfunction. Transl Neurodegener.

[19] Wrann CD, White JP, Salogiannnis J, Laznik-Bogoslavski D, Wu J, Ma D, Lin JD, Greenberg ME, Spiegelman BM (2013). Exercise induces hippocampal BDNF through a PGC-1alpha/FNDC5 pathway. Cell Metab.

[20] Liu PZ, Nusslock R (2018). Exercise-mediated neurogenesis in the hippocampus via BDNF. Front Neurosci.

[21] De Miguel Z, Khoury N, Betley MJ, Lehallier B, Willoughby D, Olsson N, Yang AC, Hahn O, Lu N, Vest RT (2021). Exercise plasma boosts memory and dampens brain inflammation via clusterin. Nature.

[22] Horowitz AM, Fan X, Bieri G, Smith LK, Sanchez-Diaz CI, Schroer AB, Gontier G, Casaletto KB, Kramer JH, Williams KE, Villeda SA (2020). Blood factors transfer beneficial effects of exercise on neurogenesis and cognition to the aged brain. Science.

[23] Sleiman SF, Henry J, Al-Haddad R, El Hayek L, Abou Haidar E, Stringer T, Ulja D, Karuppagounder SS, Holson EB, Ratan RR (2016). Exercise promotes the expression of brain derived neurotrophic factor (BDNF) through the action of the ketone body beta-hydroxybutyrate. Elife.

[24] Nigam SM, Xu S, Kritikou JS, Marosi K, Brodin L, Mattson MP (2017). Exercise and BDNF reduce Abeta production by enhancing alpha-secretase processing of APP. J Neurochem.

[25] Delezie J, Handschin C (2018). Endocrine crosstalk between skeletal muscle and the brain. Front Neurol.

[26] Pedersen BK (2019). Physical activity and muscle-brain crosstalk. Nat Rev Endocrinol.

[27] Pedersen BK, Febbraio MA (2012). Muscles, exercise and obesity: skeletal muscle as a secretory organ. Nat Rev Endocrinol.

[28] Young MF, Valaris S, Wrann CD (2019). A role for FNDC5/Irisin in the beneficial effects of exercise on the brain and in neurodegenerative diseases. Prog Cardiovasc Dis.

[29] Cao J, Zhou A, Zhou Z, Liu H, Jia S (2023). The role of GPLD1 in chronic diseases. J Cell Physiol.

[30] Beauchamp LC, Liu XM, Sedjahtera A, Bogeski M, Vella LJ, Bush AI, Adlard PA, Barnham KJ (2020). S-adenosylmethionine rescues cognitive deficits in the rTg4510 animal model by stabilizing protein phosphatase 2A and reducing phosphorylated tau. J Alzheimers Dis.

[31] Wan X, Ma B, Wang X, Guo C, Sun J, Cui J, Li L (2020). S-Adenosylmethionine alleviates amyloid-β-induced neural injury by enhancing trans-sulfuration pathway activity in astrocytes. J Alzheimers Dis.

[32] Jang YJ, Byun S (2021). Molecular targets of exercise mimetics and their natural activators. BMB Rep.

[33] Guerrieri D, Moon HY, van Praag H (2017). Exercise in a pill: the latest on exercise-mimetics. Brain Plast.

[34] Gubert C, Hannan AJ (2021). Exercise mimetics: harnessing the therapeutic effects of physical activity. Nat Rev Drug Discov.

[35] Ozben T, Ozben S (2019). Neuro-inflammation and anti-inflammatory treatment options for Alzheimer’s disease. Clin Biochem.

[36] Thinakaran G, Koo EH (2008). Amyloid precursor protein trafficking, processing, and function. J Biol Chem.

[37] Selkoe DJ (2008). Soluble oligomers of the amyloid beta-protein impair synaptic plasticity and behavior. Behav Brain Res.

[38] Panza F, Lozupone M, Seripa D, Imbimbo BP (2019). Amyloid-beta immunotherapy for Alzheimer disease: is it now a long shot?. Ann Neurol.

[39] Crystal H, Dickson D, Fuld P, Masur D, Scott R, Mehler M, Masdeu J, Kawas C, Aronson M, Wolfson L (1988). Clinico-pathologic studies in dementia: nondemented subjects with pathologically confirmed Alzheimer’s disease. Neurology.

[40] Jellinger K (2002). Prevalence of Alzheimer’s disease in very elderly people: a prospective neuropathological study. Neurology.

[41] Steubler V, Erdinger S, Back MK, Ludewig S, Fassler D, Richter M, Han K, Slomianka L, Amrein I, von Engelhardt J (2021). Loss of all three APP family members during development impairs synaptic function and plasticity, disrupts learning, and causes an autism-like phenotype. EMBO J.

[42] Sturchio A, Dwivedi AK, Young CB, Malm T, Marsili L, Sharma JS, Mahajan A, Hill EJ, Andaloussi SE, Poston KL (2021). High cerebrospinal amyloid-beta 42 is associated with normal cognition in individuals with brain amyloidosis. EClinicalMedicine.

[43] Maccioni RB, Tapia JP, Guzman-Martinez L (2018). Pathway to tau modifications and the origins of Alzheimer’s disease. Arch Med Res.

[44] Braak H, Braak E (1991). Neuropathological stageing of Alzheimer-related changes. Acta Neuropathol.

[45] Rojo LE, Fernandez JA, Maccioni AA, Jimenez JM, Maccioni RB (2008). Neuroinflammation: implications for the pathogenesis and molecular diagnosis of Alzheimer’s disease. Arch Med Res.

[46] Heneka MT, McManus RM, Latz E (2018). Inflammasome signalling in brain function and neurodegenerative disease. Nat Rev Neurosci.

[47] Leng F, Edison P (2021). Neuroinflammation and microglial activation in Alzheimer disease: where do we go from here?. Nat Rev Neurol.

[48] Li X, Shi X, McPherson M, Hager M, Garcia GG, Miller RA (2022). Cap-independent translation of GPLD1 enhances markers of brain health in long-lived mutant and drug-treated mice. Aging Cell.

[49] Huang Y, Happonen KE, Burrola PG, O'Connor C, Hah N, Huang L, Nimmerjahn A, Lemke G (2021). Microglia use TAM receptors to detect and engulf amyloid beta plaques. Nat Immunol.

[50] Maccioni RB, Rojo LE, Fernandez JA, Kuljis RO (2009). The role of neuroimmunomodulation in Alzheimer's disease. Ann NY Acad Sci.

[51] DiSabato DJ, Quan N, Godbout JP (2016). Neuroinflammation: the devil is in the details. J Neurochem.

[52] Lyman M, Lloyd DG, Ji X, Vizcaychipi MP, Ma D (2014). Neuroinflammation: the role and consequences. Neurosci Res.

[53] Gutierrez EG, Banks WA, Kastin AJ (1993). Murine tumor necrosis factor alpha is transported from blood to brain in the mouse. J Neuroimmunol.

[54] Terrando N, Eriksson LI, Ryu JK, Yang T, Monaco C, Feldmann M, Jonsson Fagerlund M, Charo IF, Akassoglou K, Maze M (2011). Resolving postoperative neuroinflammation and cognitive decline. Ann Neurol.

[55] Wong D, Dorovini-Zis K, Vincent SR (2004). Cytokines, nitric oxide, and cGMP modulate the permeability of an in vitro model of the human blood-brain barrier. Exp Neurol.

[56] Morales I, Guzman-Martinez L, Cerda-Troncoso C, Farias GA, Maccioni RB (2014). Neuroinflammation in the pathogenesis of Alzheimer’s disease. A rational framework for the search of novel therapeutic approaches. Front Cell Neurosci.

[57] Lee HJ, Kim C, Lee SJ (2010). Alpha-synuclein stimulation of astrocytes: potential role for neuroinflammation and neuroprotection. Oxid Med Cell Longev.

[58] Mann CN, Devi SS, Kersting CT, Bleem AV, Karch CM, Holtzman DM, Gallardo G (2022). Astrocytic alpha2-Na(+)/K(+) ATPase inhibition suppresses astrocyte reactivity and reduces neurodegeneration in a tauopathy mouse model. Sci Transl Med..

[59] Kwon HS, Koh SH (2020). Neuroinflammation in neurodegenerative disorders: the roles of microglia and astrocytes. Transl Neurodegener..

[60] McAlpine CS, Park J, Griciuc A, Kim E, Choi SH, Iwamoto Y, Kiss MG, Christie KA, Vinegoni C, Poller WC (2021). Astrocytic interleukin-3 programs microglia and limits Alzheimer’s disease. Nature.

[61] Ahmad MA, Kareem O, Khushtar M, Akbar M, Haque MR, Iqubal A, Haider MF, Pottoo FH, Abdulla FS, Al-Haidar MB, Alhajri N (2022). Neuroinflammation: a potential risk for dementia. Int J Mol Sci.

[62] Wyss-Coray T, Mucke L (2002). Inflammation in neurodegenerative disease–a double-edged sword. Neuron.

[63] Russo MV, McGavern DB (2016). Inflammatory neuroprotection following traumatic brain injury. Science.

[64] Anderson WD, Vadigepalli R, Jaeger D, Jung R (2022). Neuroinflammation, glia, and cytokines: networks of networks. Encyclopedia of computational neuroscience.

[65] Kempuraj D, Thangavel R, Natteru PA, Selvakumar GP, Saeed D, Zahoor H, Zaheer S, Iyer SS, Zaheer A (2016). Neuroinflammation induces neurodegeneration. J Neurol Neurosurg Spine.

[66] Mishra A, Kim HJ, Shin AH, Thayer SA (2012). Synapse loss induced by interleukin-1β requires pre- and post-synaptic mechanisms. J Neuroimmune Pharmacol.

[67] Heneka MT, Carson MJ, Khoury JE, Landreth GE, Brosseron F, Feinstein DL, Jacobs AH, Wyss-Coray T, Vitorica J, Ransohoff RM (2015). Neuroinflammation in Alzheimer’s disease. Lancet Neurol.

[68] Squillace S, Salvemini D (2022). Toll-like receptor-mediated neuroinflammation: relevance for cognitive dysfunctions. Trends Pharmacol Sci.

[69] Wen H, Miao EA, Ting JP (2013). Mechanisms of NOD-like receptor-associated inflammasome activation. Immunity.

[70] Latz E, Xiao TS, Stutz A (2013). Activation and regulation of the inflammasomes. Nat Rev Immunol.

[71] Fritz JH, Ferrero RL, Philpott DJ, Girardin SE (2006). Nod-like proteins in immunity, inflammation and disease. Nat Immunol.

[72] Platnich JM, Muruve DA (2019). NOD-like receptors and inflammasomes: a review of their canonical and non-canonical signaling pathways. Arch Biochem Biophys.

[73] Zhong Y, Kinio A, Saleh M (2013). Functions of NOD-like receptors in human diseases. Front Immunol.

[74] Sekiya M, Wang M, Fujisaki N, Sakakibara Y, Quan X, Ehrlich ME, De Jager PL, Bennett DA, Schadt EE, Gandy S (2018). Integrated biology approach reveals molecular and pathological interactions among Alzheimer’s Aβ42, Tau, TREM2, and TYROBP in Drosophila models. Genome Med.

[75] Baufeld C, O'Loughlin E, Calcagno N, Madore C, Butovsky O (2018). Differential contribution of microglia and monocytes in neurodegenerative diseases. J Neural Transm (Vienna).

[76] Dos Santos SE, Medeiros M, Porfirio J, Tavares W, Pessoa L, Grinberg L, Leite REP, Ferretti-Rebustini REL, Suemoto CK, Filho WJ (2020). Similar microglial cell densities across brain structures and mammalian species: implications for brain tissue function. J Neurosci.

[77] Nimmerjahn A, Kirchhoff F, Helmchen F (2005). Resting microglial cells are highly dynamic surveillants of brain parenchyma in vivo. Science.

[78] Davalos D, Grutzendler J, Yang G, Kim JV, Zuo Y, Jung S, Littman DR, Dustin ML, Gan WB (2005). ATP mediates rapid microglial response to local brain injury in vivo. Nat Neurosci.

[79] Stence N, Waite M, Dailey ME (2001). Dynamics of microglial activation: a confocal time-lapse analysis in hippocampal slices. Glia.

[80] Spittau B (2017). Aging microglia-phenotypes, functions and implications for age-related neurodegenerative diseases. Front Aging Neurosci.

[81] Davies DS, Ma J, Jegathees T, Goldsbury C (2017). Microglia show altered morphology and reduced arborization in human brain during aging and Alzheimer's disease. Brain Pathol.

[82] Rawji KS, Mishra MK, Michaels NJ, Rivest S, Stys PK, Yong VW (2016). Immunosenescence of microglia and macrophages: impact on the ageing central nervous system. Brain.

[83] Bisht K, Sharma KP, Lecours C, Sanchez MG, El Hajj H, Milior G, Olmos-Alonso A, Gomez-Nicola D, Luheshi G, Vallieres L (2016). Dark microglia: a new phenotype predominantly associated with pathological states. Glia.

[84] Niraula A, Sheridan JF, Godbout JP (2017). Microglia priming with aging and stress. Neuropsychopharmacology.

[85] Plescher M, Seifert G, Hansen JN, Bedner P, Steinhauser C, Halle A (2018). Plaque-dependent morphological and electrophysiological heterogeneity of microglia in an Alzheimer’s disease mouse model. Glia.

[86] Sanchez-Mejias E, Navarro V, Jimenez S, Sanchez-Mico M, Sanchez-Varo R, Nunez-Diaz C, Trujillo-Estrada L, Davila JC, Vizuete M, Gutierrez A, Vitorica J (2016). Soluble phospho-tau from Alzheimer’s disease hippocampus drives microglial degeneration. Acta Neuropathol.

[87] Doorn KJ, Goudriaan A, Blits-Huizinga C, Bol JG, Rozemuller AJ, Hoogland PV, Lucassen PJ, Drukarch B, van de Berg WD, van Dam AM (2014). Increased amoeboid microglial density in the olfactory bulb of Parkinson’s and Alzheimer's patients. Brain Pathol.

[88] Navarro V, Sanchez-Mejias E, Jimenez S, Munoz-Castro C, Sanchez-Varo R, Davila JC, Vizuete M, Gutierrez A, Vitorica J (2018). Microglia in Alzheimer’s disease: activated, dysfunctional or degenerative. Front Aging Neurosci.

[89] Varnum MM, Ikezu T (2012). The classification of microglial activation phenotypes on neurodegeneration and regeneration in Alzheimer’s disease brain. Arch Immunol Ther Exp (Warsz).

[90] Ransohoff RM (2016). A polarizing question: do M1 and M2 microglia exist?. Nat Neurosci.

[91] Colonna M, Brioschi S (2019). Neuroinflammation and neurodegeneration in human brain at single-cell resolution. Nat Rev Immunol.

[92] Keren-Shaul H, Spinrad A, Weiner A, Matcovitch-Natan O, Dvir-Szternfeld R, Ulland TK, David E, Baruch K, Lara-Astaiso D, Toth B (2017). A unique microglia type associated with restricting development of Alzheimer's disease. Cell.

[93] Ma J, Jiang T, Tan L, Yu JT (2015). TYROBP in Alzheimer’s disease. Mol Neurobiol.

[94] Galatro TF, Holtman IR, Lerario AM, Vainchtein ID, Brouwer N, Sola PR, Veras MM, Pereira TF, Leite REP, Moller T (2017). Transcriptomic analysis of purified human cortical microglia reveals age-associated changes. Nat Neurosci.

[95] Mathys H, Adaikkan C, Gao F, Young JZ, Manet E, Hemberg M, De Jager PL, Ransohoff RM, Regev A, Tsai LH (2017). Temporal tracking of microglia activation in neurodegeneration at single-cell resolution. Cell Rep.

[96] Friedman BA, Srinivasan K, Ayalon G, Meilandt WJ, Lin H, Huntley MA, Cao Y, Lee SH, Haddick PCG, Ngu H (2018). Diverse brain myeloid expression profiles reveal distinct microglial activation states and aspects of Alzheimer’s disease not evident in mouse models. Cell Rep.

[97] Zhang Y, Chen K, Sloan SA, Bennett ML, Scholze AR, O'Keeffe S, Phatnani HP, Guarnieri P, Caneda C, Ruderisch N (2014). An RNA-sequencing transcriptome and splicing database of glia, neurons, and vascular cells of the cerebral cortex. J Neurosci.

[98] McGeer PL, Itagaki S, Tago H, McGeer EG (1987). Reactive microglia in patients with senile dementia of the Alzheimer type are positive for the histocompatibility glycoprotein HLA-DR. Neurosci Lett.

[99] Tooyama I, Kimura H, Akiyama H, McGeer PL (1990). Reactive microglia express class I and class II major histocompatibility complex antigens in Alzheimer’s disease. Brain Res.

[100] Hayes A, Thaker U, Iwatsubo T, Pickering-Brown SM, Mann DM (2002). Pathological relationships between microglial cell activity and tau and amyloid beta protein in patients with Alzheimer’s disease. Neurosci Lett.

[101] Dani M, Wood M, Mizoguchi R, Fan Z, Walker Z, Morgan R, Hinz R, Biju M, Kuruvilla T, Brooks DJ, Edison P (2018). Microglial activation correlates in vivo with both tau and amyloid in Alzheimer’s disease. Brain.

[102] Del Bo R, Angeretti N, Lucca E, De Simoni MG, Forloni G (1995). Reciprocal control of inflammatory cytokines, IL-1 and IL-6, and beta-amyloid production in cultures. Neurosci Lett.

[103] Akiyama H, Barger S, Barnum S, Bradt B, Bauer J, Cole GM, Cooper NR, Eikelenboom P, Emmerling M, Fiebich BL (2000). Inflammation and Alzheimer’s disease. Neurobiol Aging.

[104] Hanisch UK (2002). Microglia as a source and target of cytokines. Glia.

[105] Yang T, Li S, Xu H, Walsh DM, Selkoe DJ (2017). Large soluble oligomers of amyloid beta-protein from Alzheimer brain are far less neuroactive than the smaller oligomers to which they dissociate. J Neurosci.

[106] Zhong L, Wang Z, Wang D, Wang Z, Martens YA, Wu L, Xu Y, Wang K, Li J, Huang R (2018). Amyloid-beta modulates microglial responses by binding to the triggering receptor expressed on myeloid cells 2 (TREM2). Mol Neurodegener.

[107] Doens D, Fernández PL (2014). Microglia receptors and their implications in the response to amyloid β for Alzheimer’s disease pathogenesis. J Neuroinflammation.

[108] Venegas C, Heneka MT (2017). Danger-associated molecular patterns in Alzheimer’s disease. J Leukoc Biol.

[109] Liu S, Liu Y, Hao W, Wolf L, Kiliaan AJ, Penke B, Rube CE, Walter J, Heneka MT, Hartmann T (2012). TLR2 is a primary receptor for Alzheimer’s amyloid beta peptide to trigger neuroinflammatory activation. J Immunol.

[110] Murgas P, Godoy B, von Bernhardi R (2012). Abeta potentiates inflammatory activation of glial cells induced by scavenger receptor ligands and inflammatory mediators in culture. Neurotox Res.

[111] Alawieyah Syed Mortadza S, Sim JA, Neubrand VE, Jiang LH (2018). A critical role of TRPM2 channel in Abeta(42) -induced microglial activation and generation of tumor necrosis factor-alpha. Glia.

[112] Husemann J, Loike JD, Kodama T, Silverstein SC (2001). Scavenger receptor class B type I (SR-BI) mediates adhesion of neonatal murine microglia to fibrillar beta-amyloid. J Neuroimmunol.

[113] White CS, Lawrence CB, Brough D, Rivers-Auty J (2017). Inflammasomes as therapeutic targets for Alzheimer’s disease. Brain Pathol.

[114] Strowig T, Henao-Mejia J, Elinav E, Flavell R (2012). Inflammasomes in health and disease. Nature.

[115] Heneka MT, Kummer MP, Stutz A, Delekate A, Schwartz S, Vieira-Saecker A, Griep A, Axt D, Remus A, Tzeng TC (2013). NLRP3 is activated in Alzheimer's disease and contributes to pathology in APP/PS1 mice. Nature.

[116] Guerreiro R, Hardy J (2014). Genetics of Alzheimer’s disease. Neurotherapeutics.

[117] Jonsson T, Stefansson H, Steinberg S, Jonsdottir I, Jonsson PV, Snaedal J, Bjornsson S, Huttenlocher J, Levey AI, Lah JJ (2013). Variant of TREM2 associated with the risk of Alzheimer’s disease. N Engl J Med.

[118] Jay TR, Miller CM, Cheng PJ, Graham LC, Bemiller S, Broihier ML, Xu G, Margevicius D, Karlo JC, Sousa GL (2015). TREM2 deficiency eliminates TREM2+ inflammatory macrophages and ameliorates pathology in Alzheimer’s disease mouse models. J Exp Med.

[119] Jay TR, von Saucken VE, Landreth GE (2017). TREM2 in Neurodegenerative diseases. Mol Neurodegener.

[120] Ulland TK, Song WM, Huang SC, Ulrich JD, Sergushichev A, Beatty WL, Loboda AA, Zhou Y, Cairns NJ, Kambal A (2017). TREM2 maintains microglial metabolic fitness in Alzheimer’s disease. Cell.

[121] Paresce DM, Chung H, Maxfield FR (1997). Slow degradation of aggregates of the Alzheimer's disease amyloid beta-protein by microglial cells. J Biol Chem.

[122] Streit WJ, Braak H, Xue Q-S, Bechmann I (2009). Dystrophic (senescent) rather than activated microglial cells are associated with tau pathology and likely precede neurodegeneration in Alzheimer’s disease. Acta Neuropathol.

[123] Raha-Chowdhury R, Henderson JW, Raha AA, Stott SRW, Vuono R, Foscarin S, Wilson L, Annus T, Fincham R, Allinson K (2018). Erythromyeloid-derived TREM2: a major determinant of Alzheimer’s disease pathology in Down Syndrome. J Alzheimers Dis.

[124] Streit WJ (2006). Microglial senescence: does the brain's immune system have an expiration date?. Trends Neurosci.

[125] Streit WJ, Sammons NW, Kuhns AJ, Sparks DL (2004). Dystrophic microglia in the aging human brain. Glia.

[126] Hawcroft G, Gardner SH, Hull MA (2003). Activation of peroxisome proliferator-activated receptor γ does not explain the antiproliferative activity of the nonsteroidal anti-inflammatory drug indomethacin on human colorectal cancer cells. J Pharmacol Exp Ther.

[127] Chen C-H, Zhou W, Liu S, Deng Y, Cai F, Tone M, Tone Y, Tong Y, Song W (2011). Increased NF-κB signalling up-regulates BACE1 expression and its therapeutic potential in Alzheimer’s disease. Int J Neuropsychopharmacol.

[128] Wang Y, Ulland TK, Ulrich JD, Song W, Tzaferis JA, Hole JT, Yuan P, Mahan TE, Shi Y, Gilfillan S (2016). TREM2-mediated early microglial response limits diffusion and toxicity of amyloid plaques. J Exp Med.

[129] Venegas C, Kumar S, Franklin BS, Dierkes T, Brinkschulte R, Tejera D, Vieira-Saecker A, Schwartz S, Santarelli F, Kummer MP (2017). Microglia-derived ASC specks cross-seed amyloid-beta in Alzheimer’s disease. Nature.

[130] Morales I, Jimenez JM, Mancilla M, Maccioni RB (2013). Tau oligomers and fibrils induce activation of microglial cells. J Alzheimers Dis.

[131] Wes PD, Easton A, Corradi J, Barten DM, Devidze N, DeCarr LB, Truong A, He A, Barrezueta NX, Polson C (2014). Tau overexpression impacts a neuroinflammation gene expression network perturbed in Alzheimer's disease. PLoS ONE.

[132] Bolos M, Llorens-Martin M, Jurado-Arjona J, Hernandez F, Rabano A, Avila J (2016). Direct evidence of internalization of tau by microglia in vitro and in vivo. J Alzheimers Dis.

[133] Asai H, Ikezu S, Tsunoda S, Medalla M, Luebke J, Haydar T, Wolozin B, Butovsky O, Kugler S, Ikezu T (2015). Depletion of microglia and inhibition of exosome synthesis halt tau propagation. Nat Neurosci.

[134] Felsky D, Roostaei T, Nho K, Risacher SL, Bradshaw EM, Petyuk V, Schneider JA, Saykin A, Bennett DA, De Jager PL (2019). Neuropathological correlates and genetic architecture of microglial activation in elderly human brain. Nat Commun.

[135] Chen W, Abud EA, Yeung ST, Lakatos A, Nassi T, Wang J, Blum D, Buee L, Poon WW, Blurton-Jones M (2016). Increased tauopathy drives microglia-mediated clearance of beta-amyloid. Acta Neuropathol Commun.

[136] Pekny M, Wilhelmsson U, Pekna M (2014). The dual role of astrocyte activation and reactive gliosis. Neurosci Lett.

[137] Liddelow SA, Barres BA (2017). Reactive astrocytes: production, function, and therapeutic potential. Immunity.

[138] Liddelow SA, Guttenplan KA, Clarke LE, Bennett FC, Bohlen CJ, Schirmer L, Bennett ML, Munch AE, Chung WS, Peterson TC (2017). Neurotoxic reactive astrocytes are induced by activated microglia. Nature.

[139] Khakh BS, Sofroniew MV (2015). Diversity of astrocyte functions and phenotypes in neural circuits. Nat Neurosci.

[140] Frost GR, Li YM (2017). The role of astrocytes in amyloid production and Alzheimer's disease. Open Biol.

[141] Thal DR, Schultz C, Dehghani F, Yamaguchi H, Braak H, Braak E (2000). Amyloid beta-protein (Abeta)-containing astrocytes are located preferentially near N-terminal-truncated Abeta deposits in the human entorhinal cortex. Acta Neuropathol.

[142] Funato H, Yoshimura M, Yamazaki T, Saido TC, Ito Y, Yokofujita J, Okeda R, Ihara Y (1998). Astrocytes containing amyloid beta-protein (Abeta)-positive granules are associated with Abeta40-positive diffuse plaques in the aged human brain. Am J Pathol.

[143] Jo S, Yarishkin O, Hwang YJ, Chun YE, Park M, Woo DH, Bae JY, Kim T, Lee J, Chun H (2014). GABA from reactive astrocytes impairs memory in mouse models of Alzheimer's disease. Nat Med.

[144] Chang J, Liu F, Lee M, Wu B, Ting K, Zara JN, Soo C, Al Hezaimi K, Zou W, Chen X (2013). NF-kappaB inhibits osteogenic differentiation of mesenchymal stem cells by promoting beta-catenin degradation. Proc Natl Acad Sci USA.

[145] Kisler K, Nelson AR, Montagne A, Zlokovic BV (2017). Cerebral blood flow regulation and neurovascular dysfunction in Alzheimer disease. Nat Rev Neurosci.

[146] Bettcher BM, Tansey MG, Dorothee G, Heneka MT (2021). Peripheral and central immune system crosstalk in Alzheimer disease—a research prospectus. Nat Rev Neurol.

[147] Heneka MT, Sastre M, Dumitrescu-Ozimek L, Dewachter I, Walter J, Klockgether T, Van Leuven F (2005). Focal glial activation coincides with increased BACE1 activation and precedes amyloid plaque deposition in APP[V717I] transgenic mice. J Neuroinflammation.

[148] Livingston G, Huntley J, Sommerlad A, Ames D, Ballard C, Banerjee S, Brayne C, Burns A, Cohen-Mansfield J, Cooper C (2020). Dementia prevention, intervention, and care: 2020 report of the Lancet Commission. Lancet.

[149] Norton S, Matthews FE, Barnes DE, Yaffe K, Brayne C (2014). Potential for primary prevention of Alzheimer’s disease: an analysis of population-based data. Lancet Neurol.

[150] Hamer M, Chida Y (2009). Physical activity and risk of neurodegenerative disease: a systematic review of prospective evidence. Psychol Med.

[151] Buchman AS, Boyle PA, Yu L, Shah RC, Wilson RS, Bennett DA (2012). Total daily physical activity and the risk of AD and cognitive decline in older adults. Neurology.

[152] Silva MVF, Loures CMG, Alves LCV, de Souza LC, Borges KBG, Carvalho MDG (2019). Alzheimer's disease: risk factors and potentially protective measures. J Biomed Sci.

[153] Brown BM, Peiffer JJ, Martins RN (2013). Multiple effects of physical activity on molecular and cognitive signs of brain aging: can exercise slow neurodegeneration and delay Alzheimer's disease?. Mol Psychiatry.

[154] Vital TM, Hernandez SSS, Pedroso RV, Teixeira CVL, Garuffi M, Stein AM, Costa JLR, Stella F (2012). Effects of weight training on cognitive functions in elderly with Alzheimer's disease. Dement Neuropsychol.

[155] Toots A, Littbrand H, Bostrom G, Hornsten C, Holmberg H, Lundin-Olsson L, Lindelof N, Nordstrom P, Gustafson Y, Rosendahl E (2017). Effects of exercise on cognitive function in older people with dementia: a randomized controlled trial. J Alzheimers Dis.

[156] Baker LD, Frank LL, Foster-Schubert K, Green PS, Wilkinson CW, McTiernan A, Plymate SR, Fishel MA, Watson GS, Cholerton BA (2010). Effects of aerobic exercise on mild cognitive impairment: a controlled trial. Arch Neurol.

[157] Weuve J, Kang JH, Manson JE, Breteler MM, Ware JH, Grodstein F (2004). Physical activity, including walking, and cognitive function in older women. JAMA.

[158] Scherder EJ, Van Paasschen J, Deijen JB, Van Der Knokke S, Orlebeke JF, Burgers I, Devriese PP, Swaab DF, Sergeant JA (2005). Physical activity and executive functions in the elderly with mild cognitive impairment. Aging Ment Health.

[159] Yu Q, Li X, Wang J, Li Y (2013). Effect of exercise training on long-term potentiation and NMDA receptor channels in rats with cerebral infarction. Exp Ther Med.

[160] Liu HL, Zhao G, Zhang H, Shi LD (2013). Long-term treadmill exercise inhibits the progression of Alzheimer's disease-like neuropathology in the hippocampus of APP/PS1 transgenic mice. Behav Brain Res.

[161] Kang EB, Kwon IS, Koo JH, Kim EJ, Kim CH, Lee J, Yang CH, Lee YI, Cho IH, Cho JY (2013). Treadmill exercise represses neuronal cell death and inflammation during Abeta-induced ER stress by regulating unfolded protein response in aged presenilin 2 mutant mice. Apoptosis.

[162] El Hayek L, Khalifeh M, Zibara V, Abi Assaad R, Emmanuel N, Karnib N, El-Ghandour R, Nasrallah P, Bilen M, Ibrahim P (2019). Lactate mediates the effects of exercise on learning and memory through SIRT1-dependent activation of hippocampal brain-derived neurotrophic factor (BDNF). J Neurosci.

[163] Baranowski BJ, Hayward GC, Marko DM, MacPherson REK (2021). Examination of BDNF treatment on BACE1 activity and acute exercise on brain BDNF signaling. Front Cell Neurosci.

[164] Scheffer DDL, Latini A (2020). Exercise-induced immune system response: anti-inflammatory status on peripheral and central organs. Biochim Biophys Acta Mol Basis Dis.

[165] Wang J, Liu S, Li G, Xiao J (2020). Exercise regulates the immune system. Adv Exp Med Biol.

[166] Mahalakshmi B, Maurya N, Lee SD, Bharath Kumar V (2020). Possible neuroprotective mechanisms of physical exercise in neurodegeneration. Int J Mol Sci.

[167] Cheng AJ, Jude B, Lanner JT (2020). Intramuscular mechanisms of overtraining. Redox Biol.

[168] Simpson RJ, Campbell JP, Gleeson M, Kruger K, Nieman DC, Pyne DB, Turner JE, Walsh NP (2020). Can exercise affect immune function to increase susceptibility to infection?. Exerc Immunol Rev.

[169] Gleeson M, Bishop NC, Stensel DJ, Lindley MR, Mastana SS, Nimmo MA (2011). The anti-inflammatory effects of exercise: mechanisms and implications for the prevention and treatment of disease. Nat Rev Immunol.

[170] Scheffer DDL, Ghisoni K, Aguiar AS, Latini A (2019). Moderate running exercise prevents excessive immune system activation. Physiol Behav.

[171] Nieman DC, Wentz LM (2019). The compelling link between physical activity and the body’s defense system. J Sport Health Sci.

[172] Metsios GS, Moe RH, Kitas GD (2020). Exercise and inflammation. Best Pract Res Clin Rheumatol.

[173] Hamer M, Sabia S, Batty GD, Shipley MJ, Tabak AG, Singh-Manoux A, Kivimaki M (2012). Physical activity and inflammatory markers over 10 years: follow-up in men and women from the Whitehall II cohort study. Circulation.

[174] Pitsavos C, Panagiotakos DB, Chrysohoou C, Kavouras S, Stefanadis C (2005). The associations between physical activity, inflammation, and coagulation markers, in people with metabolic syndrome: the ATTICA study. Eur J Cardiovasc Prev Rehabil.

[175] Kadoglou NP, Perrea D, Iliadis F, Angelopoulou N, Liapis C, Alevizos M (2007). Exercise reduces resistin and inflammatory cytokines in patients with type 2 diabetes. Diabetes Care.

[176] Timmerman KL, Flynn MG, Coen PM, Markofski MM, Pence BD (2008). Exercise training-induced lowering of inflammatory (CD14+CD16+) monocytes: a role in the anti-inflammatory influence of exercise?. J Leukoc Biol.

[177] Oliveira M, Gleeson M (2010). The influence of prolonged cycling on monocyte Toll-like receptor 2 and 4 expression in healthy men. Eur J Appl Physiol.

[178] Lancaster GI, Khan Q, Drysdale P, Wallace F, Jeukendrup AE, Drayson MT, Gleeson M (2005). The physiological regulation of toll-like receptor expression and function in humans. J Physiol.

[179] Lavin KM, Perkins RK, Jemiolo B, Raue U, Trappe SW, Trappe TA (1985). Effects of aging and lifelong aerobic exercise on basal and exercise-induced inflammation. J Appl Physiol.

[180] Nilsson MI, Bourgeois JM, Nederveen JP, Leite MR, Hettinga BP, Bujak AL, May L, Lin E, Crozier M, Rusiecki DR (2019). Lifelong aerobic exercise protects against inflammaging and cancer. PLoS ONE.

[181] Valenzuela PL, Castillo-Garcia A, Morales JS, de la Villa P, Hampel H, Emanuele E, Lista S, Lucia A (2020). Exercise benefits on Alzheimer’s disease: State-of-the-science. Ageing Res Rev.

[182] Ingold M, Tulliani N, Chan CCH, Liu KPY (2020). Cognitive function of older adults engaging in physical activity. BMC Geriatr.

[183] Gheysen F, Poppe L, DeSmet A, Swinnen S, Cardon G, De Bourdeaudhuij I, Chastin S, Fias W (2018). Physical activity to improve cognition in older adults: can physical activity programs enriched with cognitive challenges enhance the effects? A systematic review and meta-analysis. Int J Behav Nutr Phys Act.

[184] Sellami M, Gasmi M, Denham J, Hayes LD, Stratton D, Padulo J, Bragazzi N (2018). Effects of acute and chronic exercise on immunological parameters in the elderly aged: can physical activity counteract the effects of aging?. Front Immunol.

[185] Zhang SS, Zhu L, Peng Y, Zhang L, Chao FL, Jiang L, Xiao Q, Liang X, Tang J, Yang H (2022). Long-term running exercise improves cognitive function and promotes microglial glucose metabolism and morphological plasticity in the hippocampus of APP/PS1 mice. J Neuroinflammation.

[186] Jensen CS, Bahl JM, Østergaard LB, Høgh P, Wermuth L, Heslegrave A, Zetterberg H, Heegaard NHH, Hasselbalch SG, Simonsen AH (2019). Exercise as a potential modulator of inflammation in patients with Alzheimer’s disease measured in cerebrospinal fluid and plasma. Exp Gerontol.

[187] Ercan Z, Bilek F, Demir CF (2021). The effect of aerobic exercise on neurofilament light chain and glial fibrillary acidic protein level in patients with relapsing remitting type multiple sclerosis. Mult Scler Relat Disord.

[188] Mela V, Mota BC, Milner M, McGinley A, Mills KHG, Kelly AM, Lynch MA (2020). Exercise-induced re-programming of age-related metabolic changes in microglia is accompanied by a reduction in senescent cells. Brain Behav Immun.

[189] Ke HC, Huang HJ, Liang KC, Hsieh-Li HM (2011). Selective improvement of cognitive function in adult and aged APP/PS1 transgenic mice by continuous non-shock treadmill exercise. Brain Res.

[190] Zhang X, He Q, Huang T, Zhao N, Liang F, Xu B, Chen X, Li T, Bi J (2019). Treadmill exercise decreases abeta deposition and counteracts cognitive decline in APP/PS1 mice, possibly via hippocampal microglia modifications. Front Aging Neurosci.

[191] Choi DH, Kwon IS, Koo JH, Jang YC, Kang EB, Byun JE, Um HS, Park HS, Yeom DC, Cho IH, Cho JY (2014). The effect of treadmill exercise on inflammatory responses in rat model of streptozotocin-induced experimental dementia of Alzheimer’s type. J Exerc Nutrition Biochem.

[192] Koo J-H, Jang Y-C, Hwang D-J, Um H-S, Lee N-H, Jung J-H, Cho J-Y (2017). Treadmill exercise produces neuroprotective effects in a murine model of Parkinson’s disease by regulating the TLR2/MyD88/NF-κB signaling pathway. Neuroscience.

[193] Rosa JM, Camargo A, Wolin IAV, Kaster MP, Rodrigues ALS (2021). Physical exercise prevents amyloid beta (1–40)-induced disturbances in NLRP3 inflammasome pathway in the hippocampus of mice. Metab Brain Dis.

[194] Lonnemann N, Hosseini S, Marchetti C, Skouras DB, Stefanoni D, D'Alessandro A, Dinarello CA, Korte M (2020). The NLRP3 inflammasome inhibitor OLT1177 rescues cognitive impairment in a mouse model of Alzheimer's disease. Proc Natl Acad Sci USA.

[195] Zhang Y, Dong Z, Song W (2020). NLRP3 inflammasome as a novel therapeutic target for Alzheimer’s disease. Signal Transduct Target Ther.

[196] Sun L-n, Qi Js, Gao R (2018). Physical exercise reserved amyloid-beta induced brain dysfunctions by regulating hippocampal neurogenesis and inflammatory response via MAPK signaling. Brain Res.

[197] Mu Y, Gage FH (2011). Adult hippocampal neurogenesis and its role in Alzheimer’s disease. Mol Neurodegener.

[198] Babcock KR, Page JS, Fallon JR, Webb AE (2021). Adult hippocampal neurogenesis in aging and Alzheimer’s disease. Stem Cell Rep.

[199] Sung PS, Lin PY, Liu CH, Su HC, Tsai KJ (2020). Neuroinflammation and neurogenesis in Alzheimer’s disease and potential therapeutic approaches. Int J Mol Sci.

[200] Gerberding AL, Zampar S, Stazi M, Liebetanz D, Wirths O (2019). Physical activity ameliorates impaired hippocampal neurogenesis in the Tg4-42 mouse model of Alzheimer’s disease. ASN Neuro.

[201] Abshenas R, Artimani T, Shahidi S, Ranjbar A, Komaki A, Salehi I, Amiri I, Soleimani Asl S (2020). Treadmill exercise enhances the promoting effects of preconditioned stem cells on memory and neurogenesis in Abeta-induced neurotoxicity in the rats. Life Sci.

[202] Montagne A, Zhao Z, Zlokovic BV (2017). Alzheimer's disease: a matter of blood-brain barrier dysfunction?. J Exp Med.

[203] Yamazaki Y, Kanekiyo T (1965). Blood-brain barrier dysfunction and the pathogenesis of Alzheimer’s disease. Int J Mol Sci.

[204] Wang D, Chen F, Han Z, Yin Z, Ge X, Lei P (2021). Relationship between amyloid-β deposition and blood-brain barrier dysfunction in Alzheimer’s disease. Front Cell Neurosci.

[205] Profaci CP, Munji RN, Pulido RS, Daneman R (2020). The blood–brain barrier in health and disease: important unanswered questions. J Exp Med.

[206] Takata F, Nakagawa S, Matsumoto J, Dohgu S (2021). Blood-brain barrier dysfunction amplifies the development of neuroinflammation: understanding of cellular events in brain microvascular endothelial cells for prevention and treatment of BBB dysfunction. Front Cell Neurosci.

[207] Knopp RC, Banks WA, Erickson MA (2022). Physical associations of microglia and the vascular blood-brain barrier and their importance in development, health, and disease. Curr Opin Neurobiol.

[208] Małkiewicz MA, Szarmach A, Sabisz A, Cubała WJ, Szurowska E, Winklewski PJ (2019). Blood-brain barrier permeability and physical exercise. J Neuroinflammation.

[209] Bertram S, Brixius K, Brinkmann C (2016). Exercise for the diabetic brain: how physical training may help prevent dementia and Alzheimer’s disease in T2DM patients. Endocrine.

[210] Soto I, Graham LC, Richter HJ, Simeone SN, Radell JE, Grabowska W, Funkhouser WK, Howell MC, Howell GR (2015). APOE stabilization by exercise prevents aging neurovascular dysfunction and complement induction. PLoS Biol.

[211] Narkar VA, Downes M, Yu RT, Embler E, Wang YX, Banayo E, Mihaylova MM, Nelson MC, Zou Y, Juguilon H (2008). AMPK and PPARdelta agonists are exercise mimetics. Cell.

[212] Liu H-W, Chang S-J (2018). Moderate exercise suppresses NF-κB signaling and activates the SIRT1-AMPK-PGC1α axis to attenuate muscle loss in diabetic db/db mice. Front Physiol.

[213] Salminen A, Hyttinen JMT, Kaarniranta K (2011). AMP-activated protein kinase inhibits NF-κB signaling and inflammation: impact on healthspan and lifespan. J Mol Med.

[214] Shu HF, Yang T, Yu SX, Huang HD, Jiang LL, Gu JW, Kuang YQ (2014). Aerobic exercise for Parkinson's disease: a systematic review and meta-analysis of randomized controlled trials. PLoS ONE.

[215] Dauwan M, Begemann MJ, Heringa SM, Sommer IE (2016). Exercise improves clinical symptoms, quality of life, global functioning, and depression in Schizophrenia: a systematic review and meta-analysis. Schizophr Bull.

[216] Fiuza-Luces C, Santos-Lozano A, Joyner M, Carrera-Bastos P, Picazo O, Zugaza JL, Izquierdo M, Ruilope LM, Lucia A (2018). Exercise benefits in cardiovascular disease: beyond attenuation of traditional risk factors. Nat Rev Cardiol.

[217] Boa BCS, Yudkin JS, van Hinsbergh VWM, Bouskela E, Eringa EC (2017). Exercise effects on perivascular adipose tissue: endocrine and paracrine determinants of vascular function. Br J Pharmacol.

[218] Ruiz-Casado A, Martin-Ruiz A, Perez LM, Provencio M, Fiuza-Luces C, Lucia A (2017). Exercise and the hallmarks of cancer. Trends Cancer.

[219] Choi SH, Bylykbashi E, Chatila ZK, Lee SW, Pulli B, Clemenson GD, Kim E, Rompala A, Oram MK, Asselin C (2018). Combined adult neurogenesis and BDNF mimic exercise effects on cognition in an Alzheimer's mouse model. Science.

[220] Chen Y, Sun Y, Luo Z, Lin J, Qi B, Kang X, Ying C, Guo C, Yao M, Chen X (2022). Potential mechanism underlying exercise upregulated circulating blood exosome miR-215-5p to prevent necroptosis of neuronal cells and a model for early diagnosis of Alzheimer's disease. Front Aging Neurosci.

[221] Knaepen K, Goekint M, Heyman EM, Meeusen R (2010). Neuroplasticity-exercise-induced response of peripheral brain-derived neurotrophic factor: a systematic review of experimental studies in human subjects. Sports Med.

[222] Koo JH, Kwon IS, Kang EB, Lee CK, Lee NH, Kwon MG, Cho IH, Cho JY (2013). Neuroprotective effects of treadmill exercise on BDNF and PI3-K/Akt signaling pathway in the cortex of transgenic mice model of Alzheimer's disease. J Exerc Nutrition Biochem.

[223] Lu B, Nagappan G, Lu Y, Lewin GR, Carter BD (2014). BDNF and synaptic plasticity, cognitive function, and dysfunction. Neurotrophic factors, handbook of experimental pharmacology.

[224] Tapia-Arancibia L, Aliaga E, Silhol M, Arancibia S (2008). New insights into brain BDNF function in normal aging and Alzheimer disease. Brain Res Rev.

[225] Kowiański P, Lietzau G, Czuba E, Waśkow M, Steliga A, Moryś J (2017). BDNF: a key factor with multipotent impact on brain signaling and synaptic plasticity. Cell Mol Neurobiol.

[226] Gao L, Zhang Y, Sterling K, Song W (2022). Brain-derived neurotrophic factor in Alzheimer’s disease and its pharmaceutical potential. Transl Neurodegener.

[227] Ng TKS, Ho CSH, Tam WWS, Kua EH, Ho RC (2019). Decreased serum brain-derived neurotrophic factor (BDNF) levels in patients with Alzheimer’s disease (AD): a systematic review and meta-analysis. Int J Mol Sci.

[228] Mori Y, Tsuji M, Oguchi T, Kasuga K, Kimura A, Futamura A, Sugimoto A, Kasai H, Kuroda T, Yano S (2021). Serum BDNF as a potential biomarker of Alzheimer’s disease: verification through assessment of serum, cerebrospinal fluid, and medial temporal lobe atrophy. Front Neurol.

[229] Angelucci F, Spalletta G, di Iulio F, Ciaramella A, Salani F, Colantoni L, Varsi AE, Gianni W, Sancesario G, Caltagirone C, Bossu P (2010). Alzheimer’s disease (AD) and mild cognitive impairment (MCI) patients are characterized by increased BDNF serum levels. Curr Alzheimer Res.

[230] McEwen LM, Gatev EG, Jones MJ, MacIsaac JL, McAllister MM, Goulding RE, Madden KM, Dawes MG, Kobor MS, Ashe MC (2018). DNA methylation signatures in peripheral blood mononuclear cells from a lifestyle intervention for women at midlife: a pilot randomized controlled trial. Appl Physiol Nutr Metab.

[231] Nagata T, Kobayashi N, Ishii J, Shinagawa S, Nakayama R, Shibata N, Kuerban B, Ohnuma T, Kondo K, Arai H (2015). Association between DNA methylation of the BDNF promoter region and clinical presentation in Alzheimer's disease. Dement Geriatr Cogn Dis Extra.

[232] Lima Giacobbo B, Doorduin J, Klein HC, Dierckx RAJO, Bromberg E, de Vries EFJ (2018). Brain-derived neurotrophic factor in brain disorders: focus on neuroinflammation. Mol Neurobiol.

[233] Kopec BM, Zhao L, Rosa-Molinar E, Siahaan TJ (2020). Non-invasive brain delivery and efficacy of BDNF in APP/PS1 transgenic mice as a model of Alzheimer’s disease. Med Res Arch.

[234] Parrini M, Ghezzi D, Deidda G, Medrihan L, Castroflorio E, Alberti M, Baldelli P, Cancedda L, Contestabile A (2017). Aerobic exercise and a BDNF-mimetic therapy rescue learning and memory in a mouse model of Down syndrome. Sci Rep.

[235] Gao L, Tian M, Zhao H-Y, Xu Q-Q, Huang Y-M, Si Q-C, Tian Q, Wu Q-M, Hu X-M, Sun L-B (2016). TrkB activation by 7, 8-dihydroxyflavone increases synapse AMPA subunits and ameliorates spatial memory deficits in a mouse model of Alzheimer's disease. J Neurochem.

[236] Carniel BP, da Rocha NS (2021). Brain-derived neurotrophic factor (BDNF) and inflammatory markers: perspectives for the management of depression. Prog Neuropsychopharmacol Biol Psychiatry.

[237] Caviedes A, Lafourcade C, Soto C, Wyneken U (2017). BDNF/NF-kappaB signaling in the neurobiology of depression. Curr Pharm Des.

[238] Caruso GI, Spampinato SF, Costantino G, Merlo S, Sortino MA (2021). SIRT1-dependent upregulation of BDNF in human microglia challenged with Aβ: an early but transient response rescued by melatonin. Biomedicines.

[239] Ding H, Chen J, Su M, Lin Z, Zhan H, Yang F, Li W, Xie J, Huang Y, Liu X (2020). BDNF promotes activation of astrocytes and microglia contributing to neuroinflammation and mechanical allodynia in cyclophosphamide-induced cystitis. J Neuroinflammation.

[240] Poduslo JF, Curran GL (1996). Permeability at the blood-brain and blood-nerve barriers of the neurotrophic factors: NGF, CNTF, NT-3, BDNF. Brain Res Mol Brain Res.

[241] Zuccato C, Cattaneo E (2009). Brain-derived neurotrophic factor in neurodegenerative diseases. Nat Rev Neurol.

[242] Chapman CD, Frey WH, Craft S, Danielyan L, Hallschmid M, Schioth HB, Benedict C (2013). Intranasal treatment of central nervous system dysfunction in humans. Pharm Res.

[243] Kandalam S, Sindji L, Delcroix GJ, Violet F, Garric X, Andre EM, Schiller PC, Venier-Julienne MC, de Rieux A, Guicheux J, Montero-Menei CN (2017). Pharmacologically active microcarriers delivering BDNF within a hydrogel: Novel strategy for human bone marrow-derived stem cells neural/neuronal differentiation guidance and therapeutic secretome enhancement. Acta Biomater.

[244] Demikhov VG (1995). Outcomes and prognosis of diseases caused by Inkoo and Tahyna viruses. Vopr Virusol.

[245] Maak S, Norheim F, Drevon CA, Erickson HP (2021). Progress and challenges in the biology of FNDC5 and irisin. Endocr Rev.

[246] Zhao R (2022). Irisin at the crossroads of inter-organ communications: challenge and implications. Front Endocrinol.

[247] Bostrom P, Wu J, Jedrychowski MP, Korde A, Ye L, Lo JC, Rasbach KA, Bostrom EA, Choi JH, Long JZ (2012). A PGC1-alpha-dependent myokine that drives brown-fat-like development of white fat and thermogenesis. Nature.

[248] Perakakis N, Triantafyllou GA, Fernandez-Real JM, Huh JY, Park KH, Seufert J, Mantzoros CS (2017). Physiology and role of irisin in glucose homeostasis. Nat Rev Endocrinol.

[249] Wang Y, Tian M, Tan J, Pei X, Lu C, Xin Y, Deng S, Zhao F, Gao Y, Gong Y (2022). Irisin ameliorates neuroinflammation and neuronal apoptosis through integrin alphaVbeta5/AMPK signaling pathway after intracerebral hemorrhage in mice. J Neuroinflammation.

[250] Briken S, Rosenkranz SC, Keminer O, Patra S, Ketels G, Heesen C, Hellweg R, Pless O, Schulz K-H, Gold SM (2016). Effects of exercise on Irisin, BDNF and IL-6 serum levels in patients with progressive multiple sclerosis. J Neuroimmunol.

[251] Rabiee F, Lachinani L, Ghaedi S, Nasr-Esfahani MH, Megraw TL, Ghaedi K (2020). New insights into the cellular activities of Fndc5/Irisin and its signaling pathways. Cell Biosci.

[252] de Oliveira M, De Sibio MT, Mathias LS, Rodrigues BM, Sakalem ME, Nogueira CR (2020). Irisin modulates genes associated with severe coronavirus disease (COVID-19) outcome in human subcutaneous adipocytes cell culture. Mol Cell Endocrinol.

[253] Wang K, Song F, Xu K, Liu Z, Han S, Li F, Sun Y (2019). Irisin attenuates neuroinflammation and prevents the memory and cognitive deterioration in Streptozotocin-induced diabetic mice. Mediators Inflamm.

[254] Islam MR, Valaris S, Young MF, Haley EB, Luo R, Bond SF, Mazuera S, Kitchen RR, Caldarone BJ, Bettio LEB (2021). Exercise hormone irisin is a critical regulator of cognitive function. Nat Metab.

[255] Lourenco MV, Ribeiro FC, Sudo FK, Drummond C, Assunção N, Vanderborght B, Tovar-Moll F, Mattos P, De Felice FG, Ferreira ST (2020). Cerebrospinal fluid irisin correlates with amyloid-β, BDNF, and cognition in Alzheimer's disease. Alzheimers Dement.

[256] Zhang F, Hou G, Hou G, Wang C, Shi B, Zheng Y (2021). Serum irisin as a potential biomarker for cognitive decline in vascular dementia. Front Neurol.

[257] Tsai C-L, Pai M-C (2021). Circulating levels of Irisin in obese individuals at genetic risk for Alzheimer’s disease: correlations with amyloid-β, metabolic, and neurocognitive indices. Behav Brain Res.

[258] Chen K, Wang K, Wang T (2022). Protective effect of irisin against Alzheimer's disease. Front Psychiatry.

[259] Kim OY, Song J (2018). The role of irisin in Alzheimer’s disease. J Clin Med.

[260] Noda Y, Kuzuya A, Tanigawa K, Araki M, Kawai R, Ma B, Sasakura Y, Maesako M, Tashiro Y, Miyamoto M (2018). Fibronectin type III domain-containing protein 5 interacts with APP and decreases amyloid β production in Alzheimer’s disease. Mol Brain.

[261] Peng J, Deng X, Huang W, Yu J-h, Wang J-x, Wang J-p, Yang S-b, Liu X, Wang L, Zhang Y (2017). Irisin protects against neuronal injury induced by oxygen-glucose deprivation in part depends on the inhibition of ROS-NLRP3 inflammatory signaling pathway. Mol Immunol.

[262] Pignataro P, Dicarlo M, Zerlotin R, Zecca C, Dell'Abate MT, Buccoliero C, Logroscino G, Colucci S, Grano M (2021). FNDC5/Irisin system in neuroinflammation and neurodegenerative diseases: update and novel perspective. Int J Mol Sci.

[263] Hegazy MA, Abdelmonsif DA, Zeitoun TM, El-Sayed NS, Samy DM (2022). Swimming exercise versus L-carnosine supplementation for Alzheimer’s dementia in rats: implication of circulating and hippocampal FNDC5/irisin. J Physiol Biochem.

[264] Bretland KA, Lin L, Bretland KM, Smith MA, Fleming SM, Dengler-Crish CM (2021). Irisin treatment lowers levels of phosphorylated tau in the hippocampus of pre-symptomatic female but not male htau mice. Neuropathol Appl Neurobiol.

[265] Welser-Alves JV, Boroujerdi A, Tigges U, Milner R (2011). Microglia use multiple mechanisms to mediate interactions with vitronectin; non-essential roles for the highly-expressed alphavbeta3 and alphavbeta5 integrins. J Neuroinflammation.

[266] Lai S-W, Chen J-H, Lin H-Y, Liu Y-S, Tsai C-F, Chang P-C, Lu D-Y, Lin C (2018). Regulatory effects of neuroinflammatory responses through brain-derived neurotrophic factor signaling in microglial cells. Mol Neurobiol.

[267] Chen F, Swartzlander DB, Ghosh A, Fryer JD, Wang B, Zheng H (2021). Clusterin secreted from astrocyte promotes excitatory synaptic transmission and ameliorates Alzheimer’s disease neuropathology. Mol Neurodegener.

[268] Rodríguez-Rivera C, Garcia MM, Molina-Álvarez M, González-Martín C, Goicoechea C (2021). Clusterin: always protecting. Synthesis, function and potential issues. Biomed Pharmacother.

[269] Moon HJ, Herring SK, Zhao L (2021). Clusterin: a multifaceted protein in the brain. Neural Regen Res.

[270] Foster EM, Dangla-Valls A, Lovestone S, Ribe EM, Buckley NJ (2019). Clusterin in Alzheimer’s disease: mechanisms, genetics, and lessons from other pathologies. Front Neurosci.

[271] Li X, Ma Y, Wei X, Li Y, Wu H, Zhuang J, Zhao Z (2014). Clusterin in Alzheimer’s disease: a player in the biological behavior of amyloid-beta. Neurosci Bull.

[272] Yu JT, Tan L (2012). The role of clusterin in Alzheimer’s disease: pathways, pathogenesis, and therapy. Mol Neurobiol.

[273] McGeer PL, Kawamata T, Walker DG (1992). Distribution of clusterin in Alzheimer brain tissue. Brain Res.

[274] Lidstrom AM, Bogdanovic N, Hesse C, Volkman I, Davidsson P, Blennow K (1998). Clusterin (apolipoprotein J) protein levels are increased in hippocampus and in frontal cortex in Alzheimer’s disease. Exp Neurol.

[275] Nilselid AM, Davidsson P, Nagga K, Andreasen N, Fredman P, Blennow K (2006). Clusterin in cerebrospinal fluid: analysis of carbohydrates and quantification of native and glycosylated forms. Neurochem Int.

[276] Thambisetty M, Simmons A, Velayudhan L, Hye A, Campbell J, Zhang Y, Wahlund LO, Westman E, Kinsey A, Guntert A (2010). Association of plasma clusterin concentration with severity, pathology, and progression in Alzheimer disease. Arch Gen Psychiatry.

[277] Schrijvers EM, Koudstaal PJ, Hofman A, Breteler MM (2011). Plasma clusterin and the risk of Alzheimer disease. JAMA.

[278] Gupta VB, Doecke JD, Hone E, Pedrini S, Laws SM, Thambisetty M, Bush AI, Rowe CC, Villemagne VL, Ames D (2016). Plasma apolipoprotein J as a potential biomarker for Alzheimer’s disease: Australian imaging, biomarkers and lifestyle study of aging. Alzheimers Dement (Amst).

[279] Deming Y, Xia J, Cai Y, Lord J, Holmans P, Bertelsen S, Holtzman D, Morris JC, Bales K, Pickering EH (2016). A potential endophenotype for Alzheimer’s disease: cerebrospinal fluid clusterin. Neurobiol Aging.

[280] DeMattos RB, O'Dell MA, Parsadanian M, Taylor JW, Harmony JA, Bales KR, Paul SM, Aronow BJ, Holtzman DM (2002). Clusterin promotes amyloid plaque formation and is critical for neuritic toxicity in a mouse model of Alzheimer's disease. Proc Natl Acad Sci USA.

[281] Wojtas AM, Carlomagno Y, Sens JP, Kang SS, Jensen TD, Kurti A, Baker KE, Berry TJ, Phillips VR, Castanedes MC (2020). Clusterin ameliorates tau pathology in vivo by inhibiting fibril formation. Acta Neuropathol Commun.

[282] Yuste-Checa P, Trinkaus VA, Riera-Tur I, Imamoglu R, Schaller TF, Wang H, Dudanova I, Hipp MS, Bracher A, Hartl FU (2021). The extracellular chaperone clusterin enhances tau aggregate seeding in a cellular model. Nat Commun.

[283] De la Rosa A, Olaso-Gonzalez G, Arc-Chagnaud C, Millan F, Salvador-Pascual A, Garcia-Lucerga C, Blasco-Lafarga C, Garcia-Dominguez E, Carretero A, Correas AG (2020). Physical exercise in the prevention and treatment of Alzheimer’s disease. J Sport Health Sci.

[284] Wei JA, Liu L, Song X, Lin B, Cui J, Luo L, Liu Y, Li S, Li X, So KF (2023). Physical exercise modulates the microglial complement pathway in mice to relieve cortical circuitry deficits induced by mutant human TDP-43. Cell Rep.

[285] Miguel ZD, Betley MJ, Willoughby D, Lehallier B, Olsson N, Bonanno L, Fairchild KJ, Contrepois K, Elias JE, Rando TA, Wyss-Coray T (2019). Exercise conditioned plasma dampens inflammation via clusterin and boosts memory. bioRxiv.

[286] Husain MA, Laurent B, Plourde M (2021). APOE and Alzheimer’s disease: from lipid transport to physiopathology and therapeutics. Front Neurosci.

[287] Fernández-Calle R, Konings SC, Frontiñán-Rubio J, García-Revilla J, Camprubí-Ferrer L, Svensson M, Martinson I, Boza-Serrano A, Venero JL, Nielsen HM (2022). APOE in the bullseye of neurodegenerative diseases: impact of the APOE genotype in Alzheimer’s disease pathology and brain diseases. Mol Neurodegener.

[288] Navab M, Anantharamaiah GM, Reddy ST, Van Lenten BJ, Wagner AC, Hama S, Hough G, Bachini E, Garber DW, Mishra VK (2005). An oral ApoJ peptide renders HDL antiinflammatory in mice and monkeys and dramatically reduces atherosclerosis in apolipoprotein E-null mice. Arterioscler Thromb Vasc Biol.

[289] Cordero-Llana O, Scott SA, Maslen SL, Anderson JM, Boyle J, Chowhdury RR, Tyers P, Barker RA, Kelly CM, Rosser AE (2011). Clusterin secreted by astrocytes enhances neuronal differentiation from human neural precursor cells. Cell Death Differ.

[290] Charnay Y, Imhof A, Vallet PG, Kovari E, Bouras C, Giannakopoulos P (2012). Clusterin in neurological disorders: molecular perspectives and clinical relevance. Brain Res Bull.

[291] Stephan JS, Sleiman SF (2021). Exercise factors released by the liver, muscle, and bones have promising therapeutic potential for stroke. Front Neurol.

[292] Townsend LK, MacPherson REK, Wright DC (2021). New horizon: exercise and a focus on tissue-brain crosstalk. J Clin Endocrinol Metab.

[293] Maguire GA, Gossner A (1995). Glycosyl phosphatidyl inositol phospholipase D activity in human serum. Ann Clin Biochem.

[294] Metz CN, Schenkman S, Davitz MA (1991). Characterization of the plasma glycosylphosphatidylinositol-specific phospholipase D (GPI-PLD). Cell Biol Int Rep.

[295] Rhode H, Lopatta E, Schulze M, Pascual C, Schulze HP, Schubert K, Schubert H, Reinhart K, Horn A (1999). Glycosylphosphatidylinositol-specific phospholipase D in blood serum: is the liver the only source of the enzyme?. Clin Chim Acta.

[296] Wen Q, Yu-Zhen L, Ning X, Pathology DO (2014). Plasma glycoprotein phospholipase D level and its clinical significance in adults latent autoimmune diabetes. Chin Gen Pract..

[297] Hoener MC, Stieger S, Brodbeck U (1990). Isolation and characterization of a phosphatidylinositol-glycan-anchor-specific phospholipase D from bovine brain. Eur J Biochem.

[298] Lierheimer R, Kunz B, Vogt L, Savoca R, Brodbeck U, Sonderegger P (1997). The neuronal cell-adhesion molecule axonin-1 is specifically released by an endogenous glycosylphosphatidylinositol-specific phospholipase. Eur J Biochem.

[299] Abdolmaleki F, Heidarianpour A (2020). Endurance exercise training restores diabetes-induced alteration in circulating glycosylphosphatidylinositol-specific phospholipase D levels in rats. Diabetol Metab Syndr.

[300] Brown HA, Thomas PG, Lindsley CW (2017). Targeting phospholipase D in cancer, infection and neurodegenerative disorders. Nat Rev Drug Discov.

[301] Mato J, Alvarez L, Ortiz P, Pajares MA (1997). S-adenosylmethionine synthesis: molecular mechanisms and clinical implications. Pharmacol Ther.

[302] Chavez M (2000). SAMe: S-Adenosylmethionine. Am J Health Syst Pharm.

[303] Robertson KD, Wolffe AP (2000). DNA methylation in health and disease. Nat Rev Genet.

[304] Altuna M, Urdanoz-Casado A, Sanchez-Ruiz de Gordoa J, Zelaya MV, Labarga A, Lepesant JMJ, Roldan M, Blanco-Luquin I, Perdones A, Larumbe R (2019). DNA methylation signature of human hippocampus in Alzheimer’s disease is linked to neurogenesis. Clin Epigenetics.

[305] Pellegrini C, Pirazzini C, Sala C, Sambati L, Yusipov I, Kalyakulina A, Ravaioli F, Kwiatkowska KM, Durso DF, Ivanchenko M (2021). A meta-analysis of brain DNA methylation across sex, age, and Alzheimer's disease points for accelerated epigenetic aging in neurodegeneration. Front Aging Neurosci.

[306] Xie J, Xie L, Wei H, Li X-J, Lin L (2023). Dynamic regulation of DNA methylation and brain functions. Biology.

[307] Swiatowy WJ, Drzewiecka H, Kliber M, Sasiadek M, Karpinski P, Plawski A, Jagodzinski PP (2021). Physical activity and DNA methylation in humans. Int J Mol Sci.

[308] Ngwa JS, Nwulia E, Ntekim O, Bedada FB, Kwabi-Addo B, Nadarajah S, Johnson S, Southerland WM, Kwagyan J, Obisesan TO (2022). Aerobic exercise training-induced changes on DNA methylation in mild cognitively impaired elderly african americans: gene, exercise, and memory study—GEMS-I. Front Mol Neurosci.

[309] Xu M, Zhu J, Liu X-D, Luo M-Y, Xu N-J (2021). Roles of physical exercise in neurodegeneration: reversal of epigenetic clock. Transl Neurodegener.

[310] Wu C, Yang L, Tucker D, Dong Y, Zhu L, Duan R, Liu TC, Zhang Q (2018). Beneficial effects of exercise pretreatment in a sporadic Alzheimer’s rat model. Med Sci Sports Exerc.

[311] Anstee QM, Day CP (2012). S-adenosylmethionine (SAMe) therapy in liver disease: a review of current evidence and clinical utility. J Hepatol.

[312] Finkelstein JD (2007). Metabolic regulatory properties of S-adenosylmethionine and S-adenosylhomocysteine. Clin Chem Lab Med.

[313] Lu SC, Mato JM (2008). S-Adenosylmethionine in cell growth, apoptosis and liver cancer. J Gastroenterol Hepatol.

[314] Lee S, Lemere CA, Frost JL, Shea TB (2012). Dietary supplementation with S-adenosyl methionine delayed amyloid-β and tau pathology in 3xTg-AD mice. J Alzheimers Dis.

[315] Fuso A, Seminara L, Cavallaro RA, D'Anselmi F, Scarpa S (2005). S-adenosylmethionine/homocysteine cycle alterations modify DNA methylation status with consequent deregulation of PS1 and BACE and beta-amyloid production. Mol Cell Neurosci.

[316] Coppede F (2021). Epigenetic regulation in Alzheimer’s disease: is it a potential therapeutic target?. Expert Opin Ther Targets.

[317] Chan A, Tchantchou F, Graves V, Rozen R, Shea TB (2008). Dietary and genetic compromise in folate availability reduces acetylcholine, cognitive performance and increases aggression: critical role of S-adenosyl methionine. J Nutr Health Aging.

[318] Fuso A, Nicolia V, Ricceri L, Cavallaro RA, Isopi E, Mangia F, Fiorenza MT, Scarpa S (2012). S-adenosylmethionine reduces the progress of the Alzheimer-like features induced by B-vitamin deficiency in mice. Neurobiol Aging.

[319] Tchantchou F, Graves M, Ortiz D, Chan A, Rogers E, Shea TB (2006). S-adenosyl methionine: a connection between nutritional and genetic risk factors for neurodegeneration in Alzheimer’s disease. J Nutr Health Aging.

[320] Tchantchou F, Graves M, Falcone D, Shea TB (2008). S-adenosylmethionine mediates glutathione efficacy by increasing glutathione S-transferase activity: implications for S-adenosyl methionine as a neuroprotective dietary supplement. J Alzheimers Dis.

[321] Zhang Y, Ma R, Deng Q, Wang W, Cao C, Yu C, Li S, Shi L, Tian J (2023). S-adenosylmethionine improves cognitive impairment in D-galactose-induced brain aging by inhibiting oxidative stress and neuroinflammation. J Chem Neuroanat.

[322] Li Q, Cui J, Fang C, Liu M, Min G, Li L, Gong C (2017). S-Adenosylmethionine attenuates oxidative stress and neuroinflammation induced by amyloid-β through modulation of glutathione metabolism. J Alzheimers Dis.

[323] Wang W, Zhao F, Ma X, Perry G, Zhu X (2020). Mitochondria dysfunction in the pathogenesis of Alzheimer’s disease: recent advances. Mol Neurodegener.

[324] Lam AB, Kervin K, Tanis JE (2021). Vitamin B12 impacts amyloid beta-induced proteotoxicity by regulating the methionine/S-adenosylmethionine cycle. Cell Rep.

[325] Adwan L, Zawia NH (2013). Epigenetics: a novel therapeutic approach for the treatment of Alzheimer’s disease. Pharmacol Ther.

[326] Duarte FV, Palmeira CM, Rolo AP, Santulli G (2015). The emerging role of mitomirs in the pathophysiology of human disease. microRNA: medical evidence: advances in experimental medicine and biology.

[327] Chatterjee B, Sarkar M, Bose S, Alam MT, Chaudhary AA, Dixit AK, Tripathi PP, Srivastava AK (2023). MicroRNAs: key modulators of inflammation-associated diseases. Semin Cell Dev Biol.

[328] Lindsay MA (2008). microRNAs and the immune response. Trends Immunol.

[329] Nejad C, Stunden HJ, Gantier MP (2018). A guide to miRNAs in inflammation and innate immune responses. FEBS J.

[330] Tili E, Michaille J-J, Costinean S, Croce CM (2008). MicroRNAs, the immune system and rheumatic disease. Nat Clin Pract Rheumatol.

[331] Gaudet AD, Fonken LK, Watkins LR, Nelson RJ, Popovich PG (2017). MicroRNAs: roles in regulating neuroinflammation. Neuroscientist.

[332] Delay C, Mandemakers W, Hébert SS (2012). MicroRNAs in Alzheimer’s disease. Neurobiol Dis.

[333] Jaber VR, Zhao Y, Sharfman NM, Li W, Lukiw WJ (2019). Addressing Alzheimer’s disease (AD) neuropathology using anti-microRNA (AM) strategies. Mol Neurobiol.

[334] Silva FCd, Iop RdR, Andrade A, Costa VP, Gutierres Filho PJB, Silva RD (2020). Effects of physical exercise on the expression of microRNAs: a systematic review. J Strength Cond Res.

[335] Silva GJJ, Bye A, el Azzouzi H, Wisløff U (2017). MicroRNAs as important regulators of exercise adaptation. Prog Cardiovasc Dis.

[336] Xu T, Liu Q, Yao J, Dai Y, Wang H, Xiao J (2015). Circulating microRNAs in response to exercise. Scand J Med Sci Sports.

[337] Osman A (2012). MicroRNAs in health and disease—basic science and clinical applications. Clin Lab.

[338] Sayed D, Abdellatif M (2011). MicroRNAs in development and disease. Physiol Rev.

[339] Liang Y, Wang L (2021). Inflamma-microRNAs in Alzheimer’s disease: from disease pathogenesis to therapeutic potentials. Front Cell Neurosci.

[340] Walgrave H, Zhou L, De Strooper B, Salta E (2021). The promise of microRNA-based therapies in Alzheimer’s disease: challenges and perspectives. Mol Neurodegener.

[341] Lee CY, Ryu IS, Ryu J-H, Cho H-J (2021). miRNAs as therapeutic tools in Alzheimer’s Disease. Int J Mol Sci.

[342] Walgrave H, Balusu S, Snoeck S, Vanden Eynden E, Craessaerts K, Thrupp N, Wolfs L, Horre K, Fourne Y, Ronisz A (2021). Restoring miR-132 expression rescues adult hippocampal neurogenesis and memory deficits in Alzheimer's disease. Cell Stem Cell.

[343] Hansen KF, Karelina K, Sakamoto K, Wayman GA, Impey S, Obrietan K (2013). miRNA-132: a dynamic regulator of cognitive capacity. Brain Struct Funct.

[344] Salta E, Sierksma A, Vanden Eynden E, De Strooper B (2016). miR-132 loss de-represses ITPKB and aggravates amyloid and TAU pathology in Alzheimer's brain. EMBO Mol Med.

[345] Juzwik CA, Drake SS, Zhang Y, Paradis-Isler N, Sylvester A, Amar-Zifkin A, Douglas C, Morquette B, Moore CS, Fournier AE (2019). microRNA dysregulation in neurodegenerative diseases: a systematic review. Prog Neurobiol.

[346] Fan W, Liang C, Ou M, Zou T, Sun F, Zhou H, Cui L (2020). MicroRNA-146a is a wide-reaching neuroinflammatory regulator and potential treatment target in neurological diseases. Front Mol Neurosci.

[347] Taganov KD, Boldin MP, Chang KJ, Baltimore D (2006). NF-kappaB-dependent induction of microRNA miR-146, an inhibitor targeted to signaling proteins of innate immune responses. Proc Natl Acad Sci USA.

[348] Martin NA, Hyrlov KH, Elkjaer ML, Thygesen EK, Wlodarczyk A, Elbaek KJ, Aboo C, Okarmus J, Benedikz E, Reynolds R (2020). Absence of miRNA-146a differentially alters microglia function and proteome. Front Immunol.

[349] Soplinska A, Zareba L, Wicik Z, Eyileten C, Jakubik D, Siller-Matula JM, De Rosa S, Malek LA, Postula M (2020). MicroRNAs as biomarkers of systemic changes in response to endurance exercise—a comprehensive review. Diagnostics.

[350] Alipour MR, Yousefzade N, Bavil FM, Naderi R, Ghiasi R (2020). Swimming impacts on pancreatic inflammatory cytokines, miR-146a and NF-кB expression levels in type-2 diabetic rats. Curr Diabetes Rev.

[351] Mai H, Fan W, Wang Y, Cai Y, Li X, Chen F, Chen X, Yang J, Tang P, Chen H (2019). Intranasal administration of miR-146a agomir rescued the pathological process and cognitive impairment in an AD mouse model. Mol Ther Nucleic Acids.

[352] Guedes JR, Santana I, Cunha C, Duro D, Almeida MR, Cardoso AM, de Lima MC, Cardoso AL (2016). MicroRNA deregulation and chemotaxis and phagocytosis impairment in Alzheimer’s disease. Alzheimers Dement (Amst).

[353] Lukiw WJ, Alexandrov PN, Zhao Y, Hill JM, Bhattacharjee S (2012). Spreading of Alzheimer’s disease inflammatory signaling through soluble micro-RNA. NeuroReport.

[354] Guedes JR, Custodia CM, Silva RJ, de Almeida LP, de Pedroso Lima MC, Cardoso AL (2014). Early miR-155 upregulation contributes to neuroinflammation in Alzheimer's disease triple transgenic mouse model. Hum Mol Genet.

[355] Teter B, Morihara T, Lim GP, Chu T, Jones MR, Zuo X, Paul RM, Frautschy SA, Cole GM (2019). Curcumin restores innate immune Alzheimer’s disease risk gene expression to ameliorate Alzheimer pathogenesis. Neurobiol Dis.

[356] Liu D, Zhao D, Zhao Y, Wang Y, Zhao Y, Wen C (2019). Inhibition of microRNA-155 alleviates cognitive impairment in Alzheimer’s disease and involvement of neuroinflammation. Curr Alzheimer Res.

[357] Zhao J, Zhou Y, Guo M, Yue D, Chen C, Liang G, Xu L (2020). MicroRNA-7: expression and function in brain physiological and pathological processes. Cell Biosci.

[358] Feng M-G, Liu C-F, Chen L, Feng W-B, Liu M, Hai H, Lu J-M (2018). MiR-21 attenuates apoptosis-triggered by amyloid-β via modulating PDCD4/ PI3K/AKT/GSK-3β pathway in SH-SY5Y cells. Biomed Pharmacother.

[359] Long JM, Ray B, Lahiri DK (2014). MicroRNA-339-5p Down-regulates protein expression of β-site amyloid precursor protein-cleaving enzyme 1 (BACE1) in human primary brain cultures and is reduced in brain tissue specimens of Alzheimer disease subjects. J Biol Chem.

[360] Jeon SM (2016). Regulation and function of AMPK in physiology and diseases. Exp Mol Med.

[361] Jorgensen SB, Richter EA, Wojtaszewski JF (2006). Role of AMPK in skeletal muscle metabolic regulation and adaptation in relation to exercise. J Physiol.

[362] Spaulding HR, Yan Z (2022). AMPK and the adaptation to exercise. Annu Rev Physiol.

[363] Wang J, Gallagher D, DeVito LM, Cancino GI, Tsui D, He L, Keller GM, Frankland PW, Kaplan DR, Miller FD (2012). Metformin activates an atypical PKC-CBP pathway to promote neurogenesis and enhance spatial memory formation. Cell Stem Cell.

[364] van Niekerk G, Mabin T, Engelbrecht AM (2019). Anti-inflammatory mechanisms of cannabinoids: an immunometabolic perspective. Inflammopharmacology.

[365] Chen X, Li X, Zhang W, He J, Xu B, Lei B, Wang Z, Cates C, Rousselle T, Li J (2018). Activation of AMPK inhibits inflammatory response during hypoxia and reoxygenation through modulating JNK-mediated NF-kappaB pathway. Metabolism.

[366] Liu T, Zhang L, Joo D, Sun SC (2017). NF-kappaB signaling in inflammation. Signal Transduct Target Ther.

[367] Kauppinen A, Suuronen T, Ojala J, Kaarniranta K, Salminen A (2013). Antagonistic crosstalk between NF-kappaB and SIRT1 in the regulation of inflammation and metabolic disorders. Cell Signal.

[368] Yang H, Zhang W, Pan H, Feldser HG, Lainez E, Miller C, Leung S, Zhong Z, Zhao H, Sweitzer S (2012). SIRT1 activators suppress inflammatory responses through promotion of p65 deacetylation and inhibition of NF-kappaB activity. PLoS ONE.

[369] Youssef ME, Abd El-Fattah EE, Abdelhamid AM, Eissa H, El-Ahwany E, Amin NA, Hetta HF, Mahmoud MH, Batiha GE, Gobba N (2021). Interference with the AMPKalpha/mTOR/NLRP3 signaling and the IL-23/IL-17 axis effectively protects against the dextran sulfate sodium intoxication in rats: a new paradigm in empagliflozin and metformin reprofiling for the management of ulcerative colitis. Front Pharmacol.

[370] Chen MY, Ye XJ, He XH, Ouyang DY (2021). The signaling pathways regulating NLRP3 inflammasome activation. Inflammation.

[371] Seo DY, Heo JW, Ko JR, Kwak HB (2019). Exercise and neuroinflammation in health and disease. Int Neurourol J.

[372] Wang Z, van Praag H, Boecker H, Hillman CH, Scheef L, Strüder HK (2012). Exercise and the brain: neurogenesis, synaptic plasticity, spine density, and angiogenesis. Functional neuroimaging in exercise and sport sciences.

[373] Spielman LJ, Little JP, Klegeris A (2016). Physical activity and exercise attenuate neuroinflammation in neurological diseases. Brain Res Bull.

[374] Lin JY, Kuo WW, Baskaran R, Kuo CH, Chen YA, Chen WS, Ho TJ, Day CH, Mahalakshmi B, Huang CY (2020). Swimming exercise stimulates IGF1/ PI3K/Akt and AMPK/SIRT1/PGC1alpha survival signaling to suppress apoptosis and inflammation in aging hippocampus. Aging (Albany NY).

[375] Azimi M, Gharakhanlou R, Naghdi N, Khodadadi D, Heysieattalab S (2018). Moderate treadmill exercise ameliorates amyloid-β-induced learning and memory impairment, possibly via increasing AMPK activity and up-regulation of the PGC-1α/FNDC5/BDNF pathway. Peptides.

[376] Morais GP, de Sousa Neto IV, Marafon BB, Ropelle ER, Cintra DE, Pauli JR, Silva A (2023). The dual and emerging role of physical exercise-induced TFEB activation in the protection against Alzheimer's disease. J Cell Physiol.

[377] Tao L, Pashley DH (1989). The relationship between dentin bond strengths and dentin permeability. Dent Mater.

[378] Zhu Y, Sun Y, Hu J, Pan Z (2022). Insight into the mechanism of exercise preconditioning in ischemic stroke. Front Pharmacol.

[379] Palasz E, Niewiadomski W, Gasiorowska A, Wysocka A, Stepniewska A, Niewiadomska G (2019). Exercise-induced neuroprotection and recovery of motor function in animal models of Parkinson's disease. Front Neurol.

[380] Corton JM, Gillespie JG, Hawley SA, Hardie DG (1995). 5-aminoimidazole-4-carboxamide ribonucleoside. A specific method for activating AMP-activated protein kinase in intact cells?. Eur J Biochem.

[381] Sanchez J, Nozhenko Y, Palou A, Rodriguez AM (2013). Free fatty acid effects on myokine production in combination with exercise mimetics. Mol Nutr Food Res.

[382] Lauritzen HP, Brandauer J, Schjerling P, Koh HJ, Treebak JT, Hirshman MF, Galbo H, Goodyear LJ (2013). Contraction and AICAR stimulate IL-6 vesicle depletion from skeletal muscle fibers in vivo. Diabetes.

[383] Xiang H-C, Lin L-X, Hu X-F, Zhu H, Li H-P, Zhang R-Y, Hu L, Liu W-T, Zhao Y-L, Shu Y (2019). AMPK activation attenuates inflammatory pain through inhibiting NF-κB activation and IL-1β expression. J Neuroinflammation.

[384] Ayasolla KR, Giri S, Singh AK, Singh I (2005). 5-aminoimidazole-4-carboxamide-1-beta-4-ribofuranoside (AICAR) attenuates the expression of LPS- and Aβ peptide-induced inflammatory mediators in astroglia. J Neuroinflammation.

[385] He G, Zhang YW, Lee JH, Zeng SX, Wang YV, Luo Z, Dong XC, Viollet B, Wahl GM, Lu H (2014). AMP-activated protein kinase induces p53 by phosphorylating MDMX and inhibiting its activity. Mol Cell Biol.

[386] Li H, Wu J, Zhu L, Sha L, Yang S, Wei J, Ji L, Tang X, Mao K, Cao L (2018). Insulin degrading enzyme contributes to the pathology in a mixed model of Type 2 diabetes and Alzheimer’s disease: possible mechanisms of IDE in T2D and AD. Biosci Rep.

[387] Du L-L, Chai D-M, Zhao L-N, Li X-H, Zhang F-C, Zhang H-B, Liu L-B, Wu K, Liu R, Wang J-Z, Zhou X-W (2014). AMPK activation ameliorates Alzheimer's disease-like pathology and spatial memory impairment in a streptozotocin-induced Alzheimer's disease model in rats. J Alzheimers Dis.

[388] Guerrieri D, van Praag H (2015). Exercise-mimetic AICAR transiently benefits brain function. Oncotarget.

[389] Kobilo T, Guerrieri D, Zhang Y, Collica SC, Becker KG, van Praag H (2014). AMPK agonist AICAR improves cognition and motor coordination in young and aged mice. Learn Mem.

[390] Lv Z, Guo Y (2020). Metformin and its benefits for various diseases. Front Endocrinol.

[391] Vial G, Detaille D, Guigas B (2019). Role of mitochondria in the mechanism(s) of action of metformin. Front Endocrinol.

[392] Viollet B, Guigas B, Garcia NS, Leclerc J, Foretz M, Andreelli F (2011). Cellular and molecular mechanisms of metformin: an overview. Clin Sci.

[393] Khezri MR, Yousefi K, Mahboubi N, Hodaei D, Ghasemnejad-Berenji M (2022). Metformin in Alzheimer’s disease: an overview of potential mechanisms, preclinical and clinical findings. Biochem Pharmacol.

[394] Sanati M, Aminyavari S, Afshari AR, Sahebkar A (2022). Mechanistic insight into the role of metformin in Alzheimer's disease. Life Sci.

[395] Cheng JT, Huang CC, Liu IM, Tzeng TF, Chang CJ (2006). Novel mechanism for plasma glucose-lowering action of metformin in streptozotocin-induced diabetic rats. Diabetes.

[396] Matsui Y, Hirasawa Y, Sugiura T, Toyoshi T, Kyuki K, Ito M (2010). Metformin reduces body weight gain and improves glucose intolerance in high-fat diet-fed C57BL/6J mice. Biol Pharm Bull.

[397] Kim SA, Choi HC (2012). Metformin inhibits inflammatory response via AMPK-PTEN pathway in vascular smooth muscle cells. Biochem Biophys Res Commun.

[398] Kelly B, Tannahill GM, Murphy MP, O'Neill LA (2015). Metformin inhibits the production of reactive oxygen species from NADH: ubiquinone oxidoreductase to limit induction of interleukin-1beta (IL-1beta) and boosts interleukin-10 (IL-10) in lipopolysaccharide (LPS)-activated macrophages. J Biol Chem.

[399] Gu J, Ye S, Wang S, Sun W, Hu Y (2014). Metformin inhibits nuclear factor-kappaB activation and inflammatory cytokines expression induced by high glucose via adenosine monophosphate-activated protein kinase activation in rat glomerular mesangial cells in vitro. Chin Med J (Engl).

[400] Andrews M, Soto N, Arredondo M (2012). Effect of metformin on the expression of tumor necrosis factor-alpha, Toll like receptors 2/4 and C reactive protein in obese type-2 diabetic patients. Rev Med Chil.

[401] Xu X, Du C, Zheng Q, Peng L, Sun Y (2014). Effect of metformin on serum interleukin-6 levels in polycystic ovary syndrome: a systematic review. BMC Womens Health.

[402] Campbell JM, Stephenson MD, de Courten B, Chapman I, Bellman SM, Aromataris E (2018). Metformin use associated with reduced risk of dementia in patients with diabetes: a systematic review and meta-analysis. J Alzheimers Dis.

[403] Sluggett JK, Koponen M, Bell JS, Taipale H, Tanskanen A, Tiihonen J, Uusitupa M, Tolppanen AM, Hartikainen S (2020). Metformin and risk of Alzheimer’s disease among community-dwelling people with diabetes: a national case-control study. J Clin Endocrinol Metab.

[404] Hsu CC, Wahlqvist ML, Lee MS, Tsai HN (2011). Incidence of dementia is increased in type 2 diabetes and reduced by the use of sulfonylureas and metformin. J Alzheimers Dis.

[405] Luchsinger JA, Perez T, Chang H, Mehta P, Steffener J, Pradabhan G, Ichise M, Manly J, Devanand DP, Bagiella E (2016). Metformin in amnestic mild cognitive impairment: results of a pilot randomized placebo controlled clinical trial. J Alzheimers Dis.

[406] Imfeld P, Bodmer M, Jick SS, Meier CR (2012). Metformin, other antidiabetic drugs, and risk of Alzheimer's disease: a population-based case-control study. J Am Geriatr Soc.

[407] Ha J, Choi DW, Kim KJ, Cho SY, Kim H, Kim KY, Koh Y, Nam CM, Kim E (2021). Association of metformin use with Alzheimer's disease in patients with newly diagnosed type 2 diabetes: a population-based nested case-control study. Sci Rep.

[408] Tabatabaei Malazy O, Bandarian F, Qorbani M, Mohseni S, Mirsadeghi S, Peimani M, Larijani B (2022). The effect of metformin on cognitive function: a systematic review and meta-analysis. J Psychopharmacol.

[409] Bell DS (2010). Metformin-induced vitamin B12 deficiency presenting as a peripheral neuropathy. South Med J.

[410] Ng TP, Feng L, Yap KB, Lee TS, Tan CH, Winblad B (2014). Long-term metformin usage and cognitive function among older adults with diabetes. J Alzheimers Dis.

[411] Mu L, Xia D, Cai J, Gu B, Liu X, Friedman V, Liu QS, Zhao L (2022). Treadmill exercise reduces neuroinflammation, glial cell activation and improves synaptic transmission in the prefrontal cortex in 3 x Tg-AD mice. Int J Mol Sci.

[412] Lu Y, Dong Y, Tucker D, Wang R, Ahmed ME, Brann D, Zhang Q (2017). Treadmill exercise exerts neuroprotection and regulates microglial polarization and oxidative stress in a streptozotocin-induced rat model of sporadic Alzheimer's disease. J Alzheimers Dis.

[413] Leem YH, Lee YI, Son HJ, Lee SH (2011). Chronic exercise ameliorates the neuroinflammation in mice carrying NSE/htau23. Biochem Biophys Res Commun.

[414] Kohman RA, Bhattacharya TK, Wojcik E, Rhodes JS (2013). Exercise reduces activation of microglia isolated from hippocampus and brain of aged mice. J Neuroinflammation.

[415] Nichol KE, Poon WW, Parachikova AI, Cribbs DH, Glabe CG, Cotman CW (2008). Exercise alters the immune profile in Tg2576 Alzheimer mice toward a response coincident with improved cognitive performance and decreased amyloid. J Neuroinflammation.

[416] Svensson M, Andersson E, Manouchehrian O, Yang Y, Deierborg T (2020). Voluntary running does not reduce neuroinflammation or improve non-cognitive behavior in the 5xFAD mouse model of Alzheimer's disease. Sci Rep.

[417] Medhat E, Rashed L, Abdelgwad M, Aboulhoda BE, Khalifa MM, El-Din SS (2020). Exercise enhances the effectiveness of vitamin D therapy in rats with Alzheimer's disease: emphasis on oxidative stress and inflammation. Metab Brain Dis.

[418] Liu Y, Chu JMT, Yan T, Zhang Y, Chen Y, Chang RCC, Wong GTC (2020). Short-term resistance exercise inhibits neuroinflammation and attenuates neuropathological changes in 3xTg Alzheimer’s disease mice. J Neuroinflammation.

[419] Hashiguchi D, Campos HC, Wuo-Silva R, Faber J, da Gomes S, Coppi AA, Arida RM, Longo BM (2020). Resistance exercise decreases amyloid load and modulates inflammatory responses in the APP/PS1 mouse model for Alzheimer’s disease. J Alzheimers Dis.

[420] de Farias JM, Dos Santos TN, Pereira EV, de Moraes GL, Furtado BG, Tietbohl LTW, Da Costa PB, Simon KU, Muller AP (2021). Physical exercise training improves judgment and problem-solving and modulates serum biomarkers in patients with Alzheimer's disease. Mol Neurobiol.

[421] Abd El-Kader SM, Al-Jiffri OH (2016). Aerobic exercise improves quality of life, psychological well-being and systemic inflammation in subjects with Alzheimer's disease. Afr Health Sci.

[422] Tsai CL, Pai MC, Ukropec J, Ukropcova B (2019). Distinctive effects of aerobic and resistance exercise modes on neurocognitive and biochemical changes in individuals with mild cognitive impairment. Curr Alzheimer Res.

